# Vascular Implications of COVID-19: Role of Radiological Imaging, Artificial Intelligence, and Tissue Characterization: A Special Report

**DOI:** 10.3390/jcdd9080268

**Published:** 2022-08-15

**Authors:** Narendra N. Khanna, Mahesh Maindarkar, Anudeep Puvvula, Sudip Paul, Mrinalini Bhagawati, Puneet Ahluwalia, Zoltan Ruzsa, Aditya Sharma, Smiksha Munjral, Raghu Kolluri, Padukone R. Krishnan, Inder M. Singh, John R. Laird, Mostafa Fatemi, Azra Alizad, Surinder K. Dhanjil, Luca Saba, Antonella Balestrieri, Gavino Faa, Kosmas I. Paraskevas, Durga Prasanna Misra, Vikas Agarwal, Aman Sharma, Jagjit Teji, Mustafa Al-Maini, Andrew Nicolaides, Vijay Rathore, Subbaram Naidu, Kiera Liblik, Amer M. Johri, Monika Turk, David W. Sobel, Gyan Pareek, Martin Miner, Klaudija Viskovic, George Tsoulfas, Athanasios D. Protogerou, Sophie Mavrogeni, George D. Kitas, Mostafa M. Fouda, Manudeep K. Kalra, Jasjit S. Suri

**Affiliations:** 1 Department of Cardiology, Indraprastha APOLLO Hospitals, New Delhi 110001, India; 2 Stroke Monitoring and Diagnostic Division, AtheroPoint™, Roseville, CA 95661, USA; 3 Department of Biomedical Engineering, North Eastern Hill University, Shillong 793022, India; 4 Annu’s Hospitals for Skin and Diabetes, Nellore 524101, India; 5 Max Institute of Cancer Care, Max Super Specialty Hospital, New Delhi 110017, India; 6 Invasive Cardiology Division, Faculty of Medicine, University of Szeged, 6720 Szeged, Hungary; 7 Division of Cardiovascular Medicine, University of Virginia, Charlottesville, VA 22904, USA; 8 Ohio Health Heart and Vascular, Columbus, OH 43214, USA; 9 Neurology Department, Fortis Hospital, Bangalore 560076, India; 10 Heart and Vascular Institute, Adventist Health St. Helena, St Helena, CA 94574, USA; 11 Department of Physiology & Biomedical Engineering, Mayo Clinic College of Medicine and Science, Rochester, MN 55905, USA; 12 Department of Radiology, Mayo Clinic College of Medicine and Science, Rochester, MN 55905, USA; 13 Department of Radiology, Azienda Ospedaliero Universitaria, 40138 Cagliari, Italy; 14 Cardiovascular Prevention and Research Unit, Department of Pathophysiology, National & Kapodistrian University of Athens, 15772 Athens, Greece; 15 Department of Pathology, Azienda Ospedaliero Universitaria, 09124 Cagliari, Italy; 16 Department of Vascular Surgery, Central Clinic of Athens, 14122 Athens, Greece; 17 Department of Immunology, Sanjay Gandhi Postgraduate Institute of Medical Sciences, Lucknow 226014, India; 18 Ann and Robert H. Lurie Children’s Hospital of Chicago, Chicago, IL 60611, USA; 19 Allergy, Clinical Immunology and Rheumatology Institute, Toronto, ON L4Z 4C4, Canada; 20 Vascular Screening and Diagnostic Centre and University of Nicosia Medical School, 2408 Nicosia, Cyprus; 21 Nephrology Department, Kaiser Permanente, Sacramento, CA 95119, USA; 22 Electrical Engineering Department, University of Minnesota, Duluth, MN 55812, USA; 23 Department of Medicine, Division of Cardiology, Queen’s University, Kingston, ON K7L 3N6, Canada; 24 The Hanse-Wissenschaftskolleg Institute for Advanced Study, 27753 Delmenhorst, Germany; 25 Rheumatology Unit, National Kapodistrian University of Athens, 15772 Athens, Greece; 26 Minimally Invasive Urology Institute, Brown University, Providence, RI 02912, USA; 27 Men’s Health Centre, Miriam Hospital Providence, Providence, RI 02906, USA; 28 Department of Radiology and Ultrasound, University Hospital for Infectious Diseases, 10000 Zagreb, Croatia; 29 Department of Surgery, Aristoteleion University of Thessaloniki, 54124 Thessaloniki, Greece; 30 Cardiology Clinic, Onassis Cardiac Surgery Centre, 17674 Athens, Greece; 31 Academic Affairs, Dudley Group NHS Foundation Trust, Dudley DY1 2HQ, UK; 32 Arthritis Research UK Epidemiology Unit, Manchester University, Manchester M13 9PL, UK; 33 Department of Electrical and Computer Engineering, Idaho State University, Pocatello, ID 83209, USA; 34 Department of Radiology, Harvard Medical School, Boston, MA 02115, USA

**Keywords:** COVID-19, coronavirus, vascular damage, pulmonary, renal, coronary, carotid, artificial intelligence

## Abstract

The SARS-CoV-2 virus has caused a pandemic, infecting nearly 80 million people worldwide, with mortality exceeding six million. The average survival span is just 14 days from the time the symptoms become aggressive. The present study delineates the deep-driven vascular damage in the pulmonary, renal, coronary, and carotid vessels due to SARS-CoV-2. This special report addresses an important gap in the literature in understanding (i) the pathophysiology of vascular damage and the role of medical imaging in the visualization of the damage caused by SARS-CoV-2, and (ii) further understanding the severity of COVID-19 using artificial intelligence (AI)-based tissue characterization (TC). PRISMA was used to select 296 studies for AI-based TC. Radiological imaging techniques such as magnetic resonance imaging (MRI), computed tomography (CT), and ultrasound were selected for imaging of the vasculature infected by COVID-19. Four kinds of hypotheses are presented for showing the vascular damage in radiological images due to COVID-19. Three kinds of AI models, namely, machine learning, deep learning, and transfer learning, are used for TC. Further, the study presents recommendations for improving AI-based architectures for vascular studies. We conclude that the process of vascular damage due to COVID-19 has similarities across vessel types, even though it results in multi-organ dysfunction. Although the mortality rate is ~2% of those infected, the long-term effect of COVID-19 needs monitoring to avoid deaths. AI seems to be penetrating the health care industry at warp speed, and we expect to see an emerging role in patient care, reduce the mortality and morbidity rate.

## 1. Introduction

In December 2019, a case of an acute respiratory distress disease linked to the SARS-CoV-2 virus was discovered in Wuhan, China [1,2]. The infection quickly spread around the world, leading to the outbreak of a coronavirus pandemic in the year 2020. Between the dates 31 December 2019, and 1 July 2022, about 550 million instances of coronavirus disease 2019 (COVID-19) were reported all across the world, resulting in more than six million deaths [3]. COVID-19 is an acute infectious disease caused by the SARS-CoV-2 virus and is an ongoing challenge for the healthcare system worldwide [4].

Previous research has delineated that COVID-19 has extrapulmonary complications [5]. Furthermore, it was found that severe SARS-CoV-2 infection damages the endothelial layer of blood vessels, resulting in vascular dysfunction, thrombosis, and inflammation [6]. The vascular endothelium of blood vessels has active paracrine, autocrine, and endocrine roles, which are vital for (a) vascular tone regulation and vascular homeostasis inflammatory balance, (b) tight connecting barriers between cells, and (c) balancing of the thrombotic and fibrinolytic pathways [7]. Hence, endothelial dysfunction is a prime reason for evolving vascular abnormality that is characterized by vasoconstriction and plaque deposition followed by organ ischemia [8].

As part of supporting evidence, Varga et al. [9,10] observed that SARS-CoV-2 directly infected endothelial cells in several patients having comorbidities such as diabetes, hypertension, renal dysfunction, and coronary artery disease. Endothelin is the current evolving concept in COVID-19 pathophysiology, causing virus-associated vascular injury due to host immune response [11]. This principle drives the development of cytokine storms and triggers thrombotic events as well as vascular injury [9,10]. Interestingly, another study by Monteil et al. [12] on a series of patients with COVID-19 reported that SARS-CoV-2 can directly infect human blood vessels, showing the involvement of endothelial cells of vascular beds in different organs [13]. The severity of damage to these vascular beds due to COVID-19 can be characterized, which can help improve and expedite patient care [14].

Artificial Intelligence (AI) has played an important role in computer-aided diagnosis (CAD) [15,16], particularly in the classification and detection of numerous diseases [17,18,19,20]. The application of machine learning (ML) and computer-aided diagnosis [16] has recently been described and has dominated the field of medical and radiological imaging, including cardiovascular disease [21,22], liver pathologies [18], diabetes [23,24], cancers (such as thyroid [25,26], ovarian [27], prostate [28], skin [29,30]), risk characterization using carotid angiography [31,32], and coronary and vascular screening [33,34,35,36]. In addition, ML has been shown to have a strong role in the field of medical and radiological imaging techniques such as magnetic resonance imaging (MRI) [37,38], computed tomography (CT) [39], and ultrasonography (US) [40]. All of these medical imaging modalities have the ability to visualize COVID-19 lesions [37,38]. It has been demonstrated that deep learning (DL) algorithms are capable of tissue characterization (TC) of plaques, including in the carotid artery [41,42] and coronary artery [43,44,45], as well as segmentation of COVID-19-related pulmonary lesions [37,46,47]. While there have been several studies demonstrating TC of vascular damage without COVID-19, here we present a study AIbTC for risk stratification of COVID-19 disease in pulmonary, renal, coronary, and carotid arteries. As mentioned previously, AI models have been successful in forecasting disease severity [48,49,50]. Thus, we hypothesize that AIbTC systems will be effective in the future for predicting COVID-19 severity or vascular implications in the pulmonary, renal, coronary, and carotid arteries.

Thus, there is a clear need for Artificial Intelligence-based tissue characterization (AIbTC) of the vascular damage in pulmonary, renal, coronary, and carotid vessels due to COVID-19.

The layout of this study is as follows: Section 2 presents the search strategy and the statistical distribution. The route of entry of SARS-CoV-2 is shown in Section 3. The complications of COVID-19 along with the pathophysiology of vascular complications, namely, (ii-a) pulmonary, (ii-b) renal, (ii-c) coronary, and (ii-d) carotid vessels, are presented in Section 4. Section 5 presents the role of medical imaging in which AI is crucial for early diagnosis and monitoring of COVID-19-related vascular complications. Section 6 provides discussion and critical comments, followed by conclusions in Section 7.

## 2. Search Strategy

**Figure 1:**
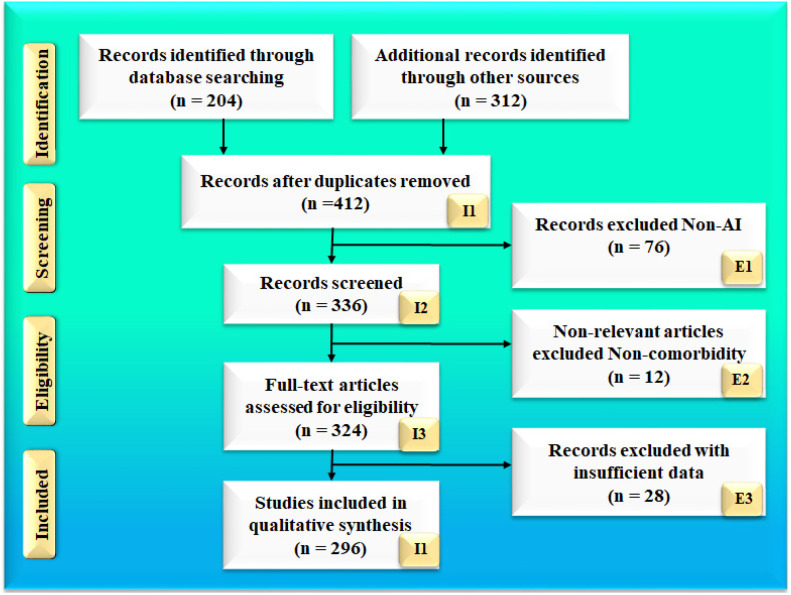
Research article search strategy; I: included, E: excluded, n: number of studies.

The search strategy followed the PRISMA methodology, which is shown in Figure 1. Two popular databases, PubMed and Google Scholar, were used to find and screen the relevant articles using the keywords“COVID-19 and vascular complications”, “coronavirus and vascular damage”, “vascular damage due to COVID-19”, “lung damage due to COVID-19”, “lung vascular complications”, “renal artery damage due to COVID-19”, “coronary artery damage due to COVID-19”, “carotid arterial damage due to COVID-19”, “artificial intelligence and vascular damage”, “tissue characterization for risk assessment”, “pathophysiology of pulmonary artery due to COVID-19”, “pathophysiology of renal artery due to COVID-19”, “pathophysiology of coronary artery due to COVID-19”, “pathophysiology of carotid artery due to COVID-19”, “pulmonary artery imaging using MR, CT, and Ultrasound”, “renal artery imaging using MR, CT, and Ultrasound”, “carotid artery imaging using MR, CT, and Ultrasound”, “coronary artery imaging using MR, CT, and Ultrasound”, “plaque tissue characterization in renal disease”, “plaque tissue characterization in pulmonary artery”, “plaque tissue characterization in coronary artery”, “plaque tissue characterization in carotid artery”, “Artificial Intelligence and renal artery”, “Artificial Intelligence and pulmonary artery”, “Artificial Intelligence and carotid artery”, “Artificial Intelligence and coronary artery”, “machine learning and renal artery”, “machine learning and pulmonary artery”, “machine learning and carotid artery”, “machine learning and coronary artery”, “deep learning and pulmonary artery”, “deep learning and renal artery”, “deep learning and carotid artery”, “deep learning and coronary artery”, “transfer learning and pulmonary artery”, “transfer learning and renal artery”, “transfer learning and carotid artery”, “transfer learning and coronary artery”. A total of 204 articles on PubMed and 312 articles on Google scholar were identified. After applying advanced filters such as time and relevance, this narrowed the search down to 336 articles. Out of these, 296 articles were screened to be included in this review. The three exclusion criteria were (i) studies not related to the topic of interest, (ii) non-relevant articles, and (iii) studies having insufficient data for analysis. This excluded 76, 12, and 28 studies (marked as E1, E2, and E3), respectively, leading to the final selection of 296 studies. The complete screening process is shown in Figure 1.

Of these, 76% (177 out of 296) of the non-AI studies included in the main manuscript related to vascular damage in the four arteries (Pulmonary, Renal, Coronary, and Carotid), and 23% were COVID-19-related, dealing with the impact of the SARS-CoV-2 virus on various organs. The focus of this manuscripts is on AI-based tissue characterization for COVID-19 severity based on vascular damage to the pulmonary, renal, coronary, and carotid arteries. The total number of AI-related studies was 119; about 68% of the studies explain AI, ML, and DL’s role in the diagnosis of vascular damage to the four arteries (Pulmonary, Renal, Coronary, and Carotid) using radiological, CT, MRI, and US imagining modalities. Even though our strategy adapted standardized engineering protocols for AI-based tissue characterization, 76% of non-AI studies were included in the manuscripts. This shows a perfect balance of AI and non-AI studies in the present study.

In Section 3, we summarize the early discoveries that contributed to the development of our knowledge of SARS-CoV-2 infection across the intracellular viral replication cycle and link it to our knowledge of coronavirus biology.

## 3. Entry Mechanism and Replication of SARS-CoV-2

**Figure 2:**
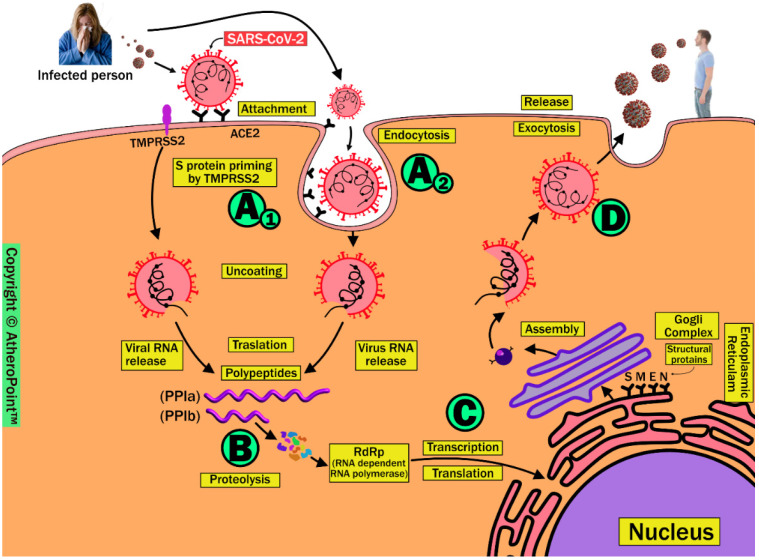
Replication of SARS-CoV-2 in four phases. (Original image, AtheroPoint™ LLC, Roseville, CA, USA).

The combination of host immune response to viral entry and high infectivity is a key factor in the wide spread of SARS-CoV-2 worldwide. It is very important to understand the molecular level changes during viral entry in order to stop SARS-CoV-2 infection from spreading. Coronaviruses (CoVs) belong to the *Nidovirales* order and *Coronaviridae* family. These are capsid membrane-enveloped, non-segmented, positive single-stranded, large genome viruses measuring approximately 30 KB. Figure 2 demonstrates the schematic replication of SARS-CoV-2 in four phases. These include (A1) attaching and uncoating, (A2) endocytosis, (B) translation and proteolysis, (C) replication and translation, and (D) assembly and exocytosis.

### 3.1. Phase A1 (Attaching and Uncoating)

Host cell entry of SARS-CoV-2 occurs due to the viral spike (S) protein attaching to the angiotensin-converting enzyme 2 receptor (ACE2) on the surface of the host cell. These proteins are the glycoproteins; the main role of glycoproteins is as support in binding and attachment of the virus to the host cell [51]. Following viral attachment, the S protein cleaves to S1 and S2 in the presence of transmembrane serine protease TMPRSS2, a process known as S protein priming. This facilitates viral fusion to the host cell membrane and allows it to enter the cytoplasm [52,53].

This phase is the target for vaccine development in blocking viral entry.

### 3.2. Phase A2 (Endocytosis)

This phase is an alternative route for SARS-CoV-2 entry into the host cell by viral translocation inside the vesicle and forming endosomes, followed by which endosomes enter the host cell. This causes virus to be released into the cytoplasm in a process called S protein priming in the presence of the endosomal cysteine proteases cathepsin B and cathepsin L. This phase of endosomal entry can be blocked by lysosomotropic agents such as hydroxychloroquine, as used in the COVID-19 preventive approach [54].

### 3.3. Phase B (Translation and Proteolysis)

After the release of the SARS-CoV-2 viral genome into the cytoplasm, it proceeds through a process called translation and forms polyproteins (pp1a and pp1ab). Subsequently, these undergo proteolysis in the presence of viral proteases and are cleaved into smaller non-structural proteins called RNA-dependent RNA polymerase (RdRP). This phase is a therapeutic target for antiviral drugs such as remdesivir, favipiravir, and ribavirin (targeting RdRP) [55,56], and lopinavir and ritonavir (protease inhibitors) [57,58].

### 3.4. Phase C (Transcription and Translation)

The viral genomic RNA in the cytoplasm is replicated by RdRP, and the structural proteins (S, E, M, and N) are translated through the endoplasmic reticulum (ER) and Golgi complex of the host cell.

### 3.5. Phase D (Assembly and Exocytosis)

Finally, these RNA genomic proteins and structural proteins assemble into novel virus particles, leading to their release through a process called exocytosis. This newly formed virus can be transmitted via salivary secretions to other individuals [52,53].

## 4. COVID-19 and Its Effect on the Vasculature

**Figure A1:**
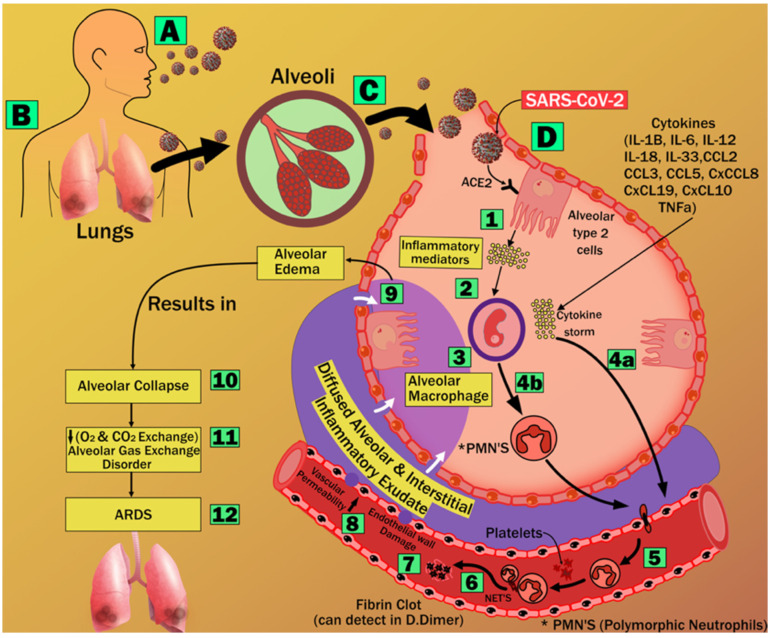
Stages of acute respiratory distress syndrome formation [296]. A: virus entry: Lung infection, C Alveoli complication, D: SARS-CoV-2 entry, 1: ACE2 binding, 2: Inflammatory mediators, 3: Alveolar Macrophage, 4a: cytokine storm, 4b: Polymorphic Neutrophils, 5: Diffuse exudate, 6: Platelets, 7:Fibrin clot, 8: Endothelial wall damage 9: Alveolar Edema, 10:Alveolar Collapse, 11: Alveolar gas exchange disorder, 12:ARDS.

In COVID-19 patients, the leading cause of mortality is hypoxia-related acute respiratory distress syndrome (ARDS) [59]. There is solid emerging evidence suggesting that damage to endothelial cells (ECs) after COVID-19 infection contributes to the initiation of ARDS (Figure A1) development [9,60]. The detailed stages of acute respiratory distress syndrome formation are shown in Appendix A.

### 4.1. Effect of COVID-19 on Pulmonary Vascular Pathology

The effects of COVID-19 on pulmonary vascular pathology are mainly due to (A) direct EC damage, (B) activation of bradykinin, (C) activation of the coagulation cascade, and (D) inflammatory response. 

#### 4.1.1. Direct Endothelial Cell Damage

SARS-CoV-2 can directly infect the ECs and cause endothelial dysfunction due to the presence of ACE2 receptors on its surface [61]. A piece of supporting evidence by Varga et al. showed the presence of viral elements within ECs, which cause inflammation and death of ECs in post-mortem analysis of admitted patients with COVID-19 positivity [9]. Further, EC death or dysfunction causes an alteration in vascular equilibrium, leading to an increase in vascular permeability.

#### 4.1.2. Activation of Bradykinin (BK)

BK is a linear non-peptide formed due to the proteolytic activity of kallikrein on kininogens, and this has strong vasopermeable and vasodilatory effects, causing angioedema [62]. Physiologically, ACE2 plays a counterbalancing role in the indirect inactivation of bradykinin by inactivating potent ligands of bradykinin-1-receptor (B1R) such as Lys des-Arg9-BK and des-Arg9-BK, in the lungs [63]. In COVID-19, SARS-CoV-2 viral entry results in the downregulation of ACE2 expression and function due to its manner of host cell entry [64]. Subsequently, reduced ACE2 levels lead to a deficiency of B1R ligand inactivators locally in the lungs, resulting in the activation of BK [65,66]. This might establish a direct link between the virus and pulmonary angioedema by causing vasodilation. Strong published pieces of evidence support this concept, such as Fu et al. [67], Glowacka et al. [68], and Levi et al. [69], all of whom have reported findings on the downregulation of ACE2 seen with SARS-CoV-1, suggesting the possibility with SARS-CoV-2 as well.

#### 4.1.3. Activation of Coagulation Cascade

Numerous publications have demonstrated that viral entry of SARS-CoV-2 occurs when the viral spike protein is anchored to the ACE2 receptor on type 2 pneumocytes on the pulmonary epithelium [70]. This triggers an innate immune response causing stimulation of macrophages followed by cytokine storm and activation of ECs [71,72]. Cytokine storm increases the expression of P-selectin, von Willebrand factor, and fibrinogen by activating ECs; platelets then bind to ECs, resulting in the initiation of the coagulation process [60]. This causes pulmonary microthrombosis, resulting in congestion and diminished ventilation response to respiratory gas exchange, followed by an increase in vascular permeability and alveolar edema [73].

#### 4.1.4. Inflammatory Process

**Figure 3:**
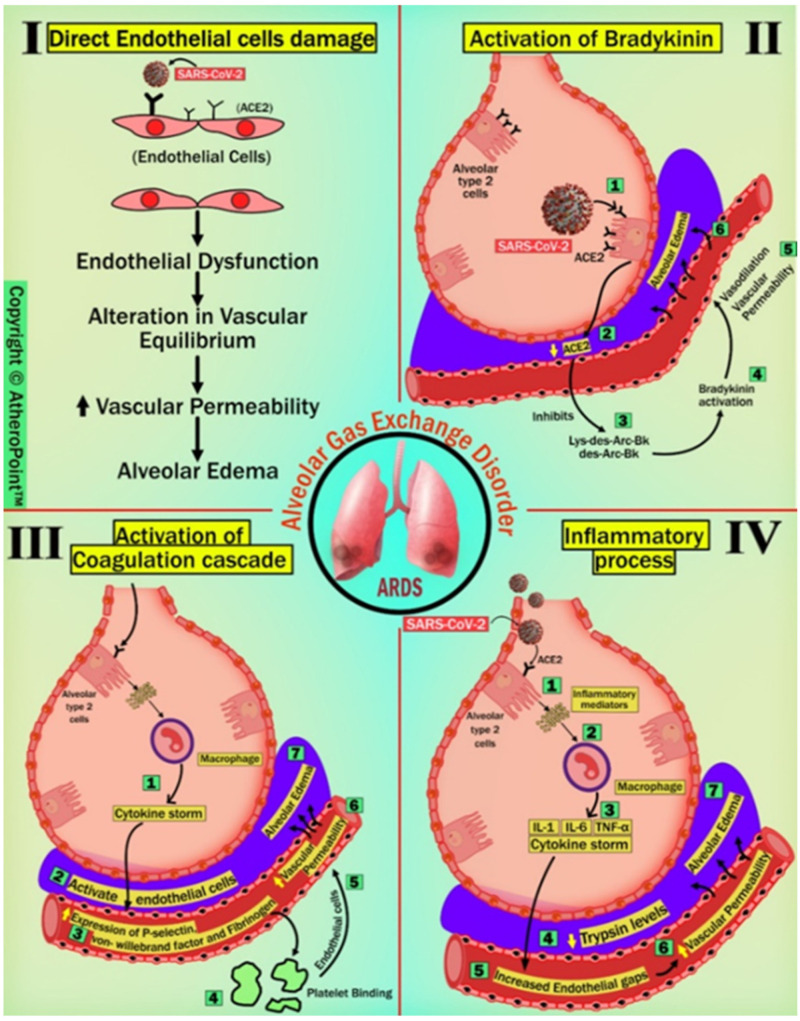
Detailed pathways of endothelial cell (EC) damage after COVID-19 infection contributing to the initiation of ARDS development. (Original image, AtheroPoint™ LLC, Roseville, CA, USA).

SARS-CoV-2 viral entry triggers a systemic inflammatory response, resulting in a cytokine storm. Cytokines such as IL-1, IL-6, and TNF- increase trypsin levels, which causes vasodilation and loosening of inter-endothelial junctions [74]. Endothelial gaps increase vascular permeability, resulting in vascular leakage and alveolar edema [60,75]. This process is similar to other members of the Corona family of viruses, such as SARS-CoV-1 and MERS-CoV [76]. As stated in the previous four sections, alveolar edema causes alveolar gas exchange disorder, which can cause the form of hypoxemia called ARDS. The detailed route information is shown diagrammatically in Figure 3.

### 4.2. Pulmonary Arterial Vascular Damage Due to COVID-19

**Figure 4:**
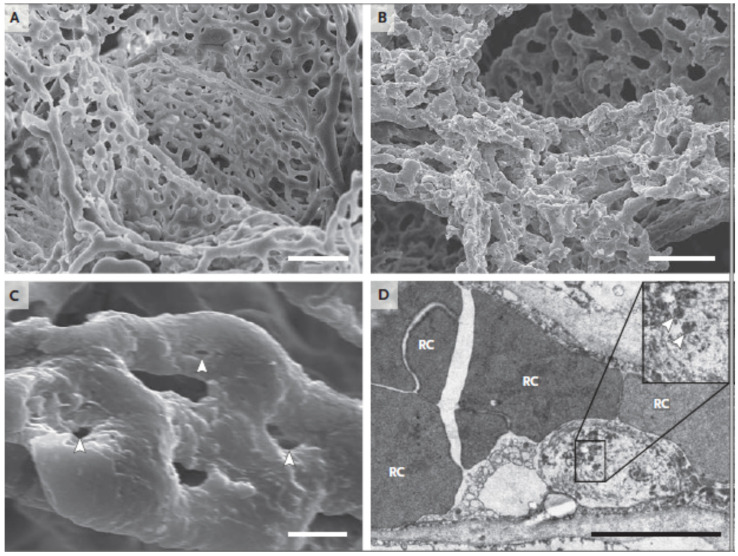
Scanning electron micrographs of (**A**) microvascular corrosion casts from the thin-walled alveolar plexus of a healthy lung and (**B**) the considerable architectural deformation seen in lungs harmed by COVID-19. In (**B**), the disappearance of a vascular hierarchy that was visible in the alveolar plexus is attributed to the development of new blood vessels via intussusceptive angiogenesis. (**C**) The intussusceptive pillar localizations at higher magnification, indicated by the arrowheads. (**D**) Transmission electron micrograph demonstrating ultrastructural aspects of the breakdown of endothelial cells and the presence of SARS-CoV-2 within the cell membrane (arrowheads). The scale bar corresponds to 5 micrometers. RC stands for red cells [77].

Pulmonary arterial vascular thrombosis has been seen in patients with confirmed COVID-19 infection. Accordingly, the morphological and molecular characteristics of seven COVID-19 patients’ lungs were retrieved after autopsies and analyzed by Ackermann et al. [77]. The lungs from these patients were compared with those retrieved through autopsies of patients who had passed away from ARDS as a result of H1N1 influenza infection and with the lungs from controls who were not infected with either virus [78]. Individuals who had influenza and patients who had COVID-19 had lungs with the same morphological pattern, which consisted of diffuse alveolar destruction and infiltrating perivascular lymphocytes. The angiocentric characteristics of COVID-19 could be broken down into three categories: (1) symptoms of significant endothelial injury linked with the intracellular SARS-CoV-2 virus and broken endothelial cell membranes [77]; (2) extensive vascular thrombosis, microangiopathy, and blockage of alveolar capillaries in the lungs of patients with COVID-19; (3) a mechanism known as intussusceptive angiogenesis was responsible for the considerable creation of new blood vessels in the lungs of individuals who had COVID-19. Even though the sample size was rather limited, the vascular abnormalities that were discovered are consistent with the occurrence of specific pulmonary vascular pathobiology features in certain cases of COVID-19 [79]. Figure 4A,B show the cases of arterial vascular thrombosis in COVID-19 patients.

A study performed by Nonno et al. [80] presented a case study of a 61-year-old woman who experienced abrupt unconsciousness and went into cardiac arrest. She was taken to an emergency department in Rome, Italy. She was unable to be revived, and her death was pronounced not long after she was brought in. ARDS and multiple organ failure are characteristics of severe instances of COVID-19, and the patient had a history of interaction with another patient who had confirmed COVID-19. The presence of a hypercoagulable disease, in conjunction with thrombosis and disseminated intravascular coagulation, has the potential to be the deciding factor in the progression to failure of multiple organs and death [81]. It has been shown that COVID-19 is linked to coagulopathies and several infarcts that have major clinical implications.

**Figure 5:**
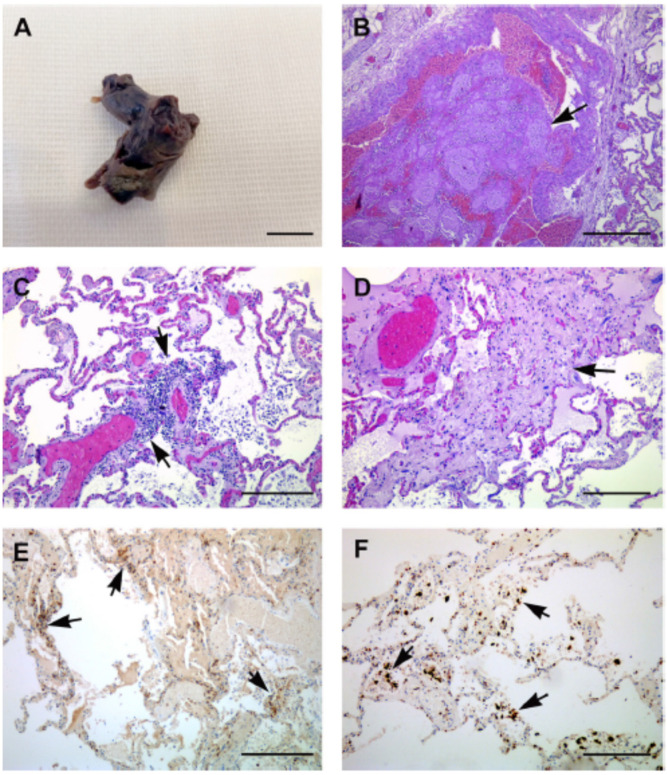
This figure shows findings related to pathology. (**A**) Gross pathological specimen of the thrombus that was obstructing both of the patient’s pulmonary arteries. The specimen is an uneven piece of hemorrhagic tissue that is reddish-tan and measures approximately 1.3 cm in diameter. (**B**) An intravascular thrombus of a major vessel can be seen in the light microscopy image of the lung tissue segment (arrow). (**C**) Inflammatory cells can be seen in the pulmonary interstitium (shown by the arrows) and in the alveolar space of the lung parenchyma. (**D**) There is evidence of widespread interstitial fibrosis in the lungs (arrow). Diffusely prevalent in the alveolar septa and around the arteries are a substantial number of CD4+ T cells (**E**) and CD68+ macrophages (**F**) (arrows). Bars on the scale read as follows: (**A**) = 1 cm; (**B**) = 100 m; (**C**–**F**) = 50 m [80].

It is evident from the cases (Figure 5) described above that COVID-19 adversely affects the arterial tissue of the pulmonary circulation. Consequently, early diagnosis using tissue characterization of patients’ pulmonary arterial condition is required in order to prevent the progression of the disease to multiple organs and reduce the risk of mortality.

#### Pulmonary CT Images

**Figure 6:**
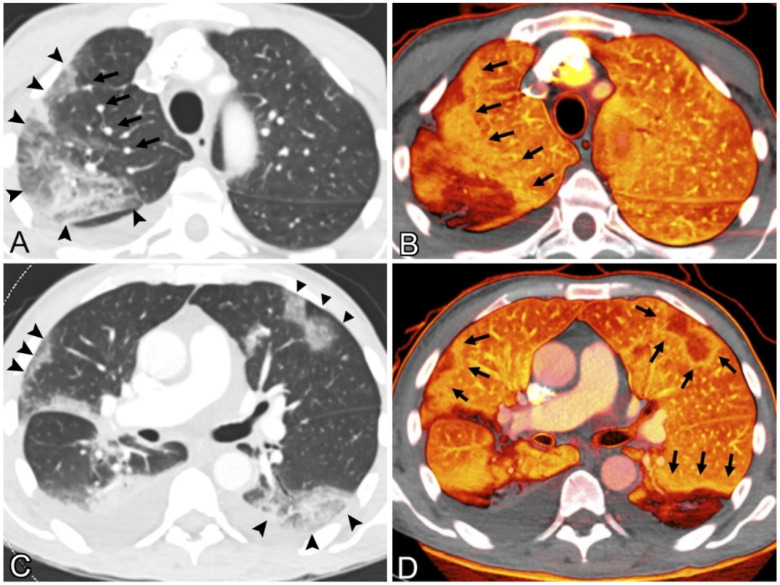
(**A**) 69-year-old man with fever, weakness, and chills had coronavirus illness. The patient was hospitalized for acute intermittent tachycardia, desaturation, and shortness of breath. No pulmonary emboli were found. Contrast-enhanced CT pulmonary angiography of the upper lungs at lung windows showed ground-glass opacity and consolidation in the right upper lobe (arrowheads); sub-segmental arteries within the opacities were dilated, and right upper lobe vessels proximal to the opacity were similarly dilated (arrows). (**B**) Pulmonary blood volume (PBV) imaging at the same level shows a significant peripheral perfusion deficiency with a surrounding halo of enhanced perfusion (arrows). Heterogeneous left upper lobe perfusion. CT scan of the patient’s lower lungs showed peripheral ground-glass opacities and consolidation with a round or wedge-shaped appearance (arrowheads). (**D**) PBV picture shows perfusion deficiencies matching the opacities in (**C**), shown with enlarged perfusion halos (arrows) [82] (2020).

Perfusion mosaics (areas of alternating higher and lower perfusion) were qualitatively assessed for mosaic perfusion, focal hyperemia (areas of the relative increase in perfusion compared with background lung), and focal oligemia (areas of the relative decrease in perfusion compared with background lung) [82]. Figure 6 shows CT images of the lung. The presence or absence of a rim around an area with low perfusion (compared to background lung) was qualitatively assessed [83]. Lung blood vessel leakage is filled with additional air sacs, resulting in shortness of breath, and can lead to ARDS.

The development of ARDS has been seen in up to 41% of patients hospitalized for COVID-19, and in 20% of COVID-19 pneumonia cases. Conversely, individuals who demonstrate reasonably preserved lung compliance may require intubation, which suggests the inclusion of additional processes in parenchymal injury [84]. Recent investigations have revealed that a loss of perfusion regulation and a loss of normal physiological hypoxic vasoconstriction contributes to the hypoxemia that is seen in individuals with COVID-19. The results of this pulmonary research are presented in Table 1.

### 4.3. Effect of COVID-19 in Renal Vascular Pathology

**Figure 7:**
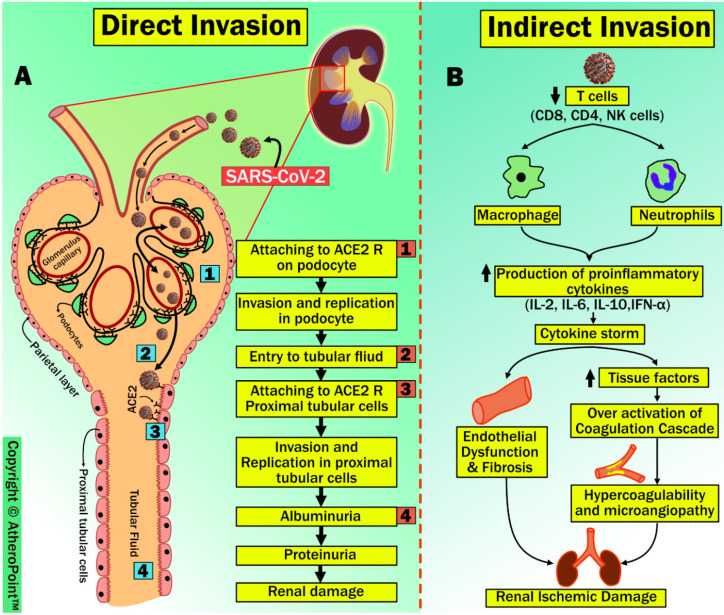
Renal vascular damage due to COVID-19 through direct and indirect invasion. (Original image, AtheroPoint™ LLC, Roseville, CA, USA) (**A**): Direct Invasion, (**B**): Indirect Invasion.

Other than targeting the alveolar epithelium, as shown in Section 2, SARS-CoV-2 attaches to ACE2 receptors present in the kidney, mainly in the tubular epithelium of proximal tubules, afferent arterioles, and collecting ducts. Additionally, it has been previously demonstrated that viral nucleic acid is found in the urine, suggesting that the kidneys are one of the targets of SARS-CoV-2 [85]. Supporting evidence by Zou et al. [86], and Pan et al. [87] shows that the kidneys are more vulnerable to SARS-CoV-2 by stratifying human organs with high or low expression of ACE2 receptors. In this section, we postulate two different pathophysiological routes by which SARS-CoV-2 affects renal tissues: (1) direct renal invasion, and (2) indirect renal invasion (shown in Figure 7).

#### 4.3.1. Direct Renal Invasion

A noteworthy observation made by Diao et al. [88] and Su et al. [89] has shown the presence of SARS-CoV-2 nucleocapsid protein and viral particles in tubular structures, podocytes, and tubular epithelial cells of the kidney. Both these solid pieces of evidence suggest that direct renal invasion exists due to SARS-CoV-2. The sequence of steps in this process includes: (1) the viral spike protein attaches to the ACE2 receptor, and the TMPRSS gene on the surface of renal epithelial cells aids viral invasion and replication in podocytes; (2) it enters the tubular fluid; (3) it binds to ACE2 receptors on the apical brush border of proximal tubular cells of the kidney, resulting in viral invasion and replication in proximal tubular cells [90]; (4) this causes albuminuria and proteinuria, leading to acute tubular necrosis [91]. However, a more data-based study is needed to support this process.

**Table 1:** The effect of COVID-19 on Pulmonary.

SN	Citations	PS	ME	Comorbidities	Outcome	Vascular Damage	Imaging Modalities	Treatment
1	Hasan et al. [92] (2020)	NR	LBBM	NR	COVID-19—A vascular disease	COVID-19—A vascular disease	CT	NR
2	Lang et al. [82] (2020)	45	LBBM	Cancer	In COVID-19 pneumonia, pulmonary vascular anomalies such as vessel hypertrophy and regional mosaic perfusion patterns are frequent.	Pulmonary vascular dilatation can occur not just within lung opacities, but also in a regional pattern outside of parenchymal opacities, and it can even affect the subpleural lung.	CT	NR
3	Ackermann et al. [93] (2020)	07	LBBM	Hypertension	The greater degree of endothelialitis and thrombosis in the lungs of patients with intussusceptive angiogenesis observed in these patients may have contributed to tissue hypoxia in both groups of patients.	Endothelialitis and thrombosis in the lung	CT	NR
4	Lins et al. [94] (2020)	NR	LBBM	NR	COVID-19 etiology involves pulmonary hemodynamic changes in the lung.	The pulmonary arterial density and tiny blood vessel volume were determined.	CT	NR
5	Hékimian et al. [95] (2020)	51	LBBM	Hypertension	Pulmonary infarction and agenesis observed	Pulmonary infarction	CT	NR
6	Espallargas et al. [96] (2020)	804	LBBM	NR	Elevation of D-dimer increases the risk of pulmonary embolism	It is possible that PE primarily affects the segmental arteries and the right lung in COVID-19 individuals.	CT	NR
7	Kho et al. [97] (2020)	15	LBBM	Dyspnoea	Radiological characteristics of COVID-19 included traction bronchiectasis, organising pneumonia, airspace opacification, inter/intra-lobular septal thickening, and bilateral peripheral subpleural ground-glass opacities.	Ground-glass opacities and areas of consolidation near the base	CT	NR
8	Miró et al. [98] (2021)	62	LBBM	CVD, Hypertension	D-dimer increases risk of pulmonary embolism	Pulmonary infarction	CT	NR
9	Scholkmann et al. [99] (2021)	01	LBBM	NR	Although focal vessel enlargement within ground-glass opacities was described in early imaging investigations of COVID-19, we have noted additional extensive vascular abnormalities.	vasculopathy was a direct viral effect on endothelial cells or perivascular inflammation	CT	NR
10	Faggiano et al. [100] (2021)	07	LBBM	CVD	COVID-19 increased risk of venous thromboembolism	COVID-19 pneumonia D-dimer levels frequently rise two to three-fold	CT	NR

PS: Patient size, ME: Method of Evaluation, CVD: Cardiovascular Disease, LBBM: Laboratory base biomarker, NR: Not reported, CT: Computer Tomography.

#### 4.3.2. Indirect Renal Invasion

Many studies performed on the pathophysiology of acute kidney injury (AKI) due to SARS-CoV-2 infection show the possibility of direct damage by viral invasion and replication as well as indirectly through cellular damage due to inflammatory response [88,101]. In this process, patients with SARS-CoV-2 infection show marked lymphopenia, mainly due to a significant reduction in T cell counts (i.e., CD8, CD4, and NK lymphocytes) [102]. Simultaneously, increased activation of neutrophils and macrophages results in the secretion of proinflammatory cytokines [103], particularly, high levels of interleukins (IL) IL-2, IL-6, IL-10, and interferon (IFN)- γ generated due to inflammatory response in a recognized as a cytokine storm [104]. In general, T cells are responsible for diminishing the effects of an overactive innate immune response during any viral infection [105]. As per this hypothesis, Lagunas-Rangel et al. have shown that reduced T cell levels results in increased concentrations of proinflammatory cytokines after COVID-19 infection. Furthermore, the same study demonstrated that increased IL-6/IFN- γ is due to cytokine storms [106]. Cytokine storms associated with inflammatory response can result in renal failure due to endothelial dysfunction and fibrosis [101]. Conversely, cytokine storms can lead to a hypercoagulability state due to the release of tissue factors and the activation of coagulation factors. This hypercoagulability state favors microangiopathies, which weaken renal perfusion, in turn leading to renal ischemia and cortical necrosis [90]. Recent clinical autopsy reports from China and the United States have confirmed that the cause of microangiopathy in several organs is due to hypercoagulability after SARS-CoV-2 infection [107,108].

Studies such as those included in Table 2 indicate that the kidney, with its high concentration of cellular ACE2 receptors, is a likely viral target. The glomerulus, mesangial cells, podocytes, and distal nephron are the primary cellular structures in which these receptors are found. In diabetic renal patients, researchers found that reactive oxygen species (ROS), kidney fibrosis, collagen deposition, mesangial matrix expansion, and podocyte loss were all present. In addition, infection with COVID-19 has been linked to anomalies in coagulation and to complement-mediated extensive thrombotic microvascular damage [109]. These patients were found to have high readings of D-dimer, fibrin degradation product, and fibrinogen, as well as an elevated international normalized ratio, normal values for partial thromboplastin time, and normal platelet count values [110].

#### 4.3.3. CT Images of the Renal Artery

**Figure 8:**
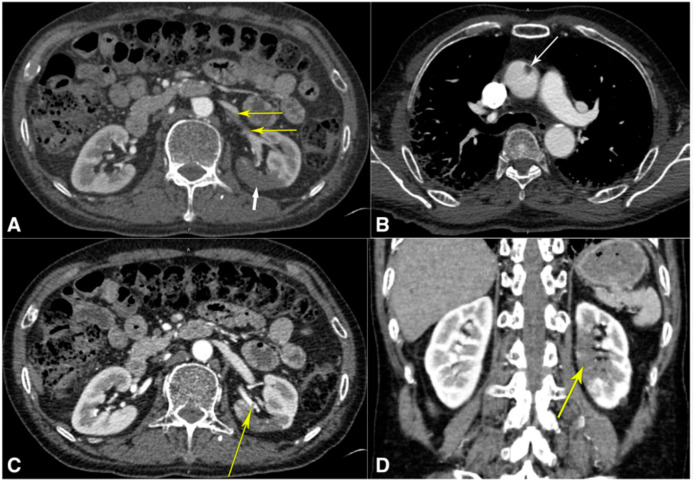
Tomography computed using angiography: (**A**) abdominal computed tomography angiography (CTA), demonstrating thrombi in the left superior renal artery (thin yellow arrows) and infarcts in the posterior mid-pole of the left kidney (thick white arrow); (**B**) CTA of the thorax, demonstrating ascending aortic thrombus (arrow); (**C**) abdominal CTA displaying a different perspective of the left superior renal artery thrombus (yellow arrow); (**D**) computed tomography abdominal angiography in coronal projection, demonstrating the extent of the left renal infarction (yellow arrow). This image is presented in color at www.ajmh.org (accessed on 28 March 2020). Mukherjee et al. [121].

In the present study, it was observed that severe COVID-19 pneumonia was responsible for inducing a prothrombotic condition, which ultimately led to ascending aortic thrombosis. This thrombus most likely dislodged itself, although it is possible that the renal artery experienced an isolated neothrombosis concurrently [111]. This thromboembolic disorder was characterized by renal infarction as its primary symptom. The case of renal artery thrombi is shown below in Figure 8.

**Table 2:** The effect of COVID-19 on renal artery.

SN	Citations	PS	ME	Comorbidities	Outcome	Vascular Damage	Imaging Modalities	Treatment
1	Acharya et al. [112] (2020)	01	LBBM	CVD	Necrosis of renal artery due to COVID-19	Renal Thrombosis	US	NR
2	Philipponnet et al. [113] (2020)	01	LBBM	CVD, Diabetics	Due to increased inflammation, platelet activation, endothelial dysfunction, and stasis, COVID-19 may predispose individuals to thrombotic illness in both the venous and arterial circulation.	Renal Thrombosis	CT	NR
3	Gabarre et al. [114] (2020)	116	LBBM	Hypertension, Diabetics	Direct invasion of SARS-CoV-2 into the renal parenchyma, an unbalanced RAAS, and micro thrombosis lead to kidney disease.	Renal Thrombosis	MRI	immunomodulatory drugs, anticoagulation
4	Yarijani et al. [115] (2020)	NR	LBBM	Diabetes mellitus, hypertension	SARS-CoV-2 enters in kidney and destroys cells, disrupting the renin–angiotensin–aldosterone system balance, activating coagulation pathways, and damaging the renal vascular endothelium are all effects of the COVID-19.	Acute Kidney Damage	US	Remdesivir, Doxycycline, Azithromycin, Chloroquine and hydroxychloroquine, Favipiravir
5	Singh et al. [116] (2020)	01	LBBM	Mucormycosis	Renal artery thrombosis in a COVID-19 patient led to renal infarction and nephrectomy.	Renal Artery Infarction	US	Anticoagulation
6	El Shamy et al. [117] (2021)	01	LBBM	Hypertension	Bilateral renal artery thrombosis due to COVID-19.	Renal Thrombosis	US	Kidney replacement
7	Watchorn et al. [118] (2021)	03	LBBM	Hypertension, CVD	Disrupting the renin–angiotensin–aldosterone system balance	Renal Thrombosis	US	NR
8	Tancredi et al. [119] (2021)	01	LBBM	Diabetes, Asthma	The observations shows that loss of corticomedullary differentiation, increased resistive indices, and decreased Doppler flow, renal cortical echogenicity increased.	Renal Artery Infarction	US	NR
9	Lushina et al. [120] (2021)	01	LBBM	Hypertension	RAAS and microthrombosis leads to CKD.	Renal Thromboembolic	CT	NR
10	Sifaat et al. [84] (2022)	NR	LBBM	Hypertension, Diabetics	The kidney is a likely target for COVID-19 due to its high number of cellular ACE2 receptors. These receptors are mainly localized in the glomerulus, mesangial cells, podocytes, and distal nephron. Reactive oxygen species (ROS), kidney fibrosis, collagen deposition, mesangial matrix expansion and podocyte loss were observed in diabetic renal disease	Kidney Fibrosis	US	RAAS antagonists

PS: Patient size, ME: Method of Evaluation, CVD: Cardiovascular Disease, LBBM: Laboratory base biomarker, NR: Not reported, CT: Computer Tomography, US: Ultrasound, MRI: Magnetic Resonance Imaging.

### 4.4. Effect of COVID-19 on Coronary/Carotid Vascular Pathology

**Figure 9:**
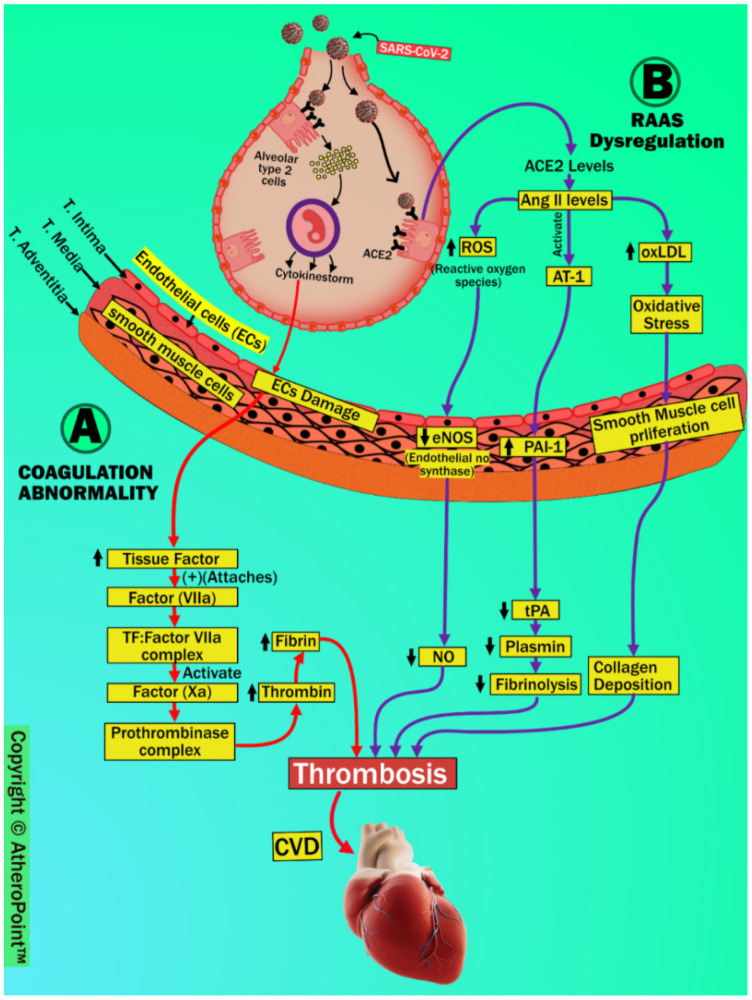
COVID-19-induced cardiovascular implications. (Original image, AtheroPoint™ LLC, Roseville, CA, USA) (**A**): Coagulation Abnormality, (**B**): RAAS Dysregulation.

In the preceding sections, we have explained the possible pathophysiology of pulmonary and renal vascular involvement in COVID-19 patients; in this section, we discuss the coronary artery. Several reports have suggested a strong relationship between COVID-19 and cardiovascular (CV) complications [59,122]. SARS-CoV-2 is linked to CVD due to thrombosis and thromboembolic events, mainly because of coagulation abnormalities and RAAS dysregulation, as shown in Figure 9.

#### 4.4.1. Coagulation Abnormality

COVID-19 induces a cytokine surge or storm, which causes vascular injury and initiates a coagulation cascade via a severe inflammatory response and endothelial barrier disruption [72,123,124].

Damaged endothelial cells upregulate tissue factors (TF) and attach to the circulating serine protease coagulation Factor VII (Factor VIIa). Further, this arrangement results in the formation of the TF: Factor VIIa complex, which then activates Factor Xa. This stimulation forms the prothrombinase complex by binding to factor Va in the presence of calcium and the phospholipid membrane [125]. These steps result in the development of thrombin and cause the recruitment of platelets. This contributes to the formation of fibrin and promotes plaque formation [126,127].

#### 4.4.2. (B) RAAS Dysregulation

The renin-angiotensin-aldosterone system (RAAS) is a complex hormonal axis which controls blood pressure, sodium absorption, and plaque formation [128,129]. Angiotensin (Ang) II is the primary physiological product of RAAS, and its functions show unpleasant effects on the human body. Furthermore, as a counterbalancing role, Ang II breaks down to Ang 1-7 via catalyzation of ACE2. After SAR-CoV-2 infection, significantly reduced ACE2 levels lead to dysregulation of RAAS and cause increased Ang II levels [130]. Increased Ang II levels result in the embellishment of functions such as vasoconstriction, increased production of cytokines, and induced organ damage [129,131,132]. In addition to vasoconstriction and cytokine production, it causes several harmful effects on the vascular wall. This is largely due to its action on the angiotensin II type 1 (AT1) receptor [133]. Higher levels of endothelial Ang II trigger the production of reactive oxygen species (ROS) and result in the breakdown of nitric oxide (NO) production by reducing endothelial nitric oxide synthase (eNOS).

This process favors endothelial dysfunction, resulting in atherosclerosis [134]. Additionally, increased Ang II levels promote atherogenesis by upregulating endothelial receptors for oxidized low-density lipoprotein (OxLDL) production. This results in oxidative stress, leading to smooth muscle cell proliferation and collagen deposition in the vessel wall, causing narrowing of the vascular lumen [135]. Moreover, increased Ang II levels via activation of angiotensin 1 receptor (AT1) upregulate plasminogen activator type 1 (PAI-1) and downregulate tissue plasminogen activator (tPA) [136]. Increased PAI-1 and decreased tPA are associated with thrombus formation due to reduced plasmin levels and fibrinolysis [137].

Collectively, activation of the coagulation cascade and increased Ang II levels due to RAAS dysregulation are major contributing factors to the development of cardiovascular events after SARS-CoV-2 infection. According to the findings shown in Table 3, individuals with COVID-19 have a much greater incidence of cardiovascular comorbidities, which puts them at an increased risk of morbidity and mortality. In COVID-19, it is suggested that clinically justified patients continue taking drugs that contain ACE inhibitors and ARBs.

### 4.5. Coronary and Carotid Artery Images

**Figure 10:**
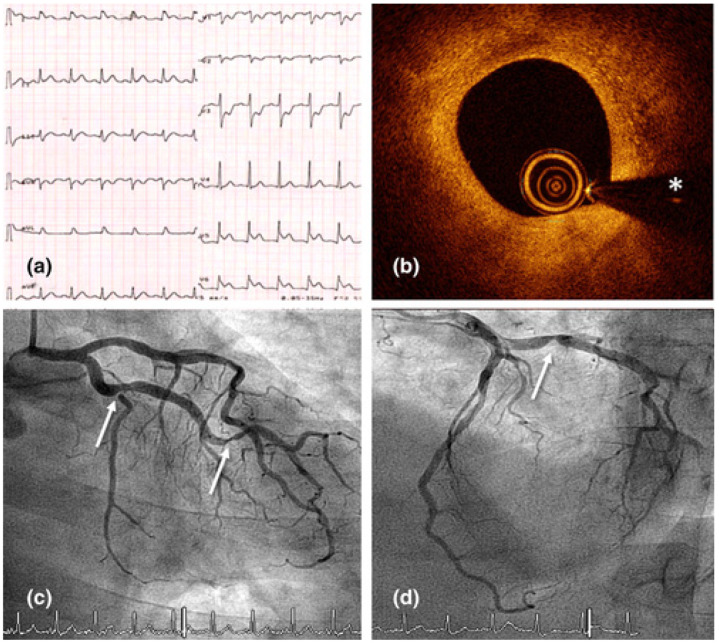
(**a**) An electrocardiogram shows inferolateral ST-segment elevation and specular decline in right precordial leads during a chest pain episode; (**b**) an intracoronary OCT image of the proximal left circumflex coronary artery (LCX) shows a stable fibrous plaque with a minimal lumen area. Erosion or rupture as an ACS cause was ruled out (asterisk denotes wire artifact). (**c**,**d**) Urgent coronary angiography demonstrating proximal and distal LCX lesions [144].

The COVID-19 virus has been linked to acute coronary syndrome in several investigations as well as case reports [144]. Researchers in Italy reported a study of 28 patients with verified COVID-19 who had undergone a coronary angiogram for diagnosis of ST-elevation myocardial infarction. Eighty-six percent of these patients had an ST-elevation myocardial infarction as the initial presentation of COVID-19. Seventy-nine percent of these patients presented with normal chest pain, while twenty-one percent did so with dyspnea in the absence of any chest pain. This shows that COVID-19 was responsible for acute coronary syndrome (ACS) even though there was not a significant amount of inflammation throughout the body [145]. Figure 10 shows an electrocardiogram that confirms inferolateral ST-segment elevation and specular decline in right precordial leads during chest pain episodes.

Myocardial hyperinflammation can lead to acute coronary syndrome, myocarditis, heart rate variability, heart failure, cardiac arrhythmias, and even unexpected death [146]. The early stages of COVID-19 are characterized by a high level of cardiac troponins and natriuretic peptides, which is indicative of acute damage to the myocardium. Acute coronary syndrome, myocarditis, heart failure, cardiac arrhythmias, and sudden death are among the potential outcomes of hyperinflammation in the myocardium.

**Figure 11:**
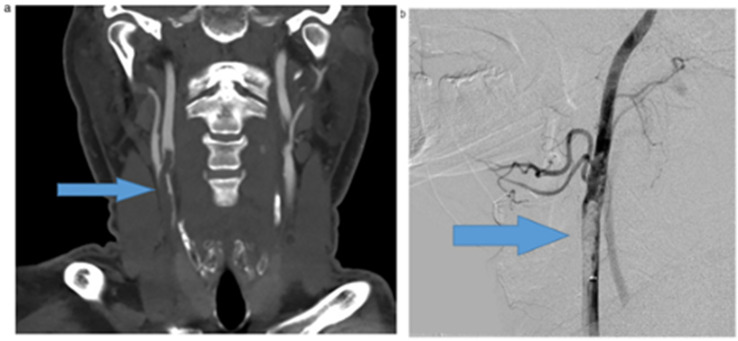
A significant amount of thrombus in the carotid artery. A man in his 50s who went to the doctor complaining of weakness in his left wrist was found to have positive serology for COVID-19. A significant subocclusive thrombosis of the right common carotid artery that extended into the internal and external carotid arteries was seen on the head and neck (**a**) CT angiography (arrows). The CT perfusion analysis revealed an acute infarct in the right superior frontal lobe as well as a wide area of elevated Tmax in the right cerebral hemisphere which involved both the right frontal and parietal lobes, indicating an area that may be at risk for additional infarction (box). (**b**) Immediately afterwards, endovascular chemical thrombolysis of the right carotid artery was carried out [144].

**Figure 12:**
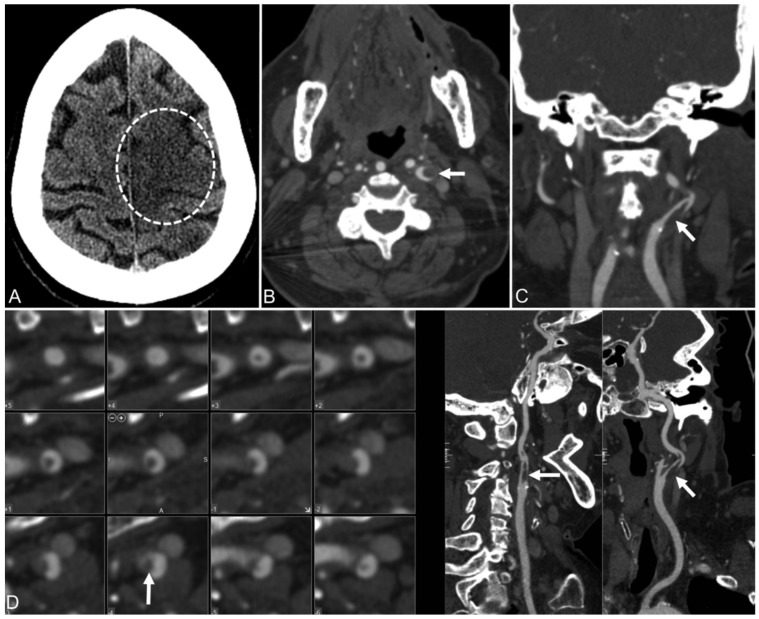
Patient 2. (**A**) 78-year-old woman with COVID-19 and an NIHSS score of 25.The CT of the head without comparison shows an evolving ischemic infarct in the left frontal brain paracentral cortex (dotted circle) and a smaller infarct in the left parietal cortex. (**B**–**D**), Axial, coronal, and curved reimaged images from CT angiography of the head and neck show an irregular plaque at the left internal carotid artery bifurcation and a capillary filling defect (arrow) extending superiorly in the left internal carotid artery, which matches the ruptured plaque with clot formation [144].

An acute myocardial injury is indicated by the high levels of cardiac troponins and natriuretic peptides which are present early on in the course of COVID-19. Table 4 shows studies related to the effect of COVID-19 on carotid vascular damage. Figure 11 and Figure 12 show a significant amount of thrombus in the carotid artery. A man in his 50s who went to the doctor complaining of weakness in his left wrist was found to have positive serology for COVID-19.

An uneven plaque at the left internal carotid artery bifurcation and an intraluminal filling defect in the left internal carotid artery, which corresponds to the ruptured plaque with clot development, can be seen in the CT angiography of the head and neck [144].

## 5. Role of Artificial Intelligence-Based Tissue Characterization

AI has played a vital role in the vascular management of COVID-19 patients. This section highlights the role of AI in the management of four different organs using the vasculature components. Section 5.1, Section 5.2, Section 5.3, Section 5.3.1, Section 5.3.2, Section 5.3.3 and Section 5.3.4 discuss the role of AI in pulmonary vessels, renal artery disease, and coronary and carotid arterial disease, respectively, in the presence of COVID-19.

**Figure 13:**
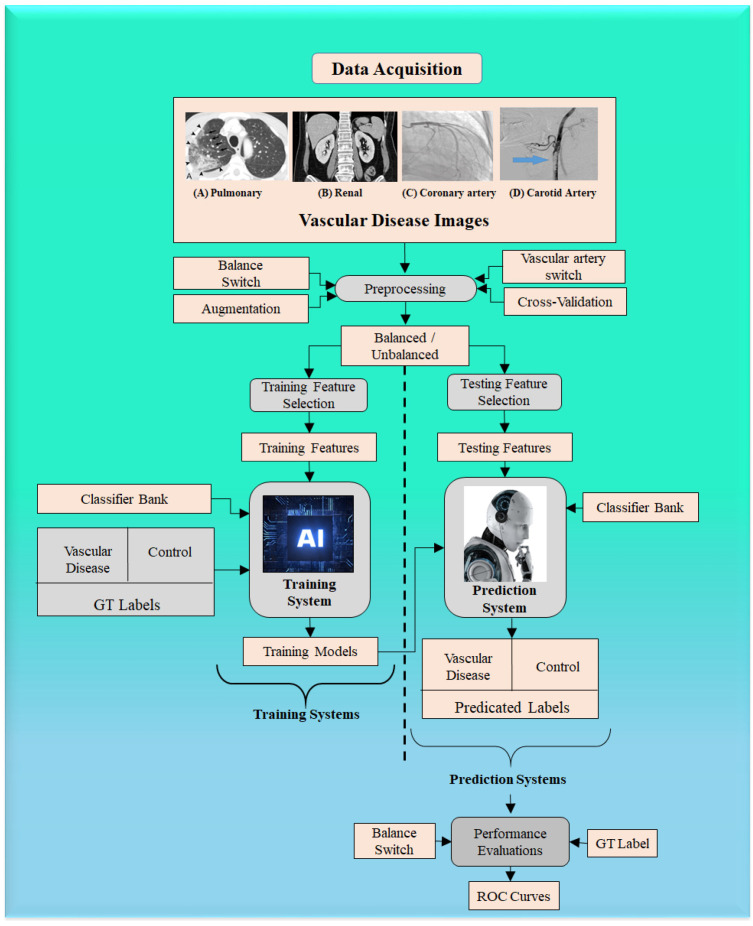
Machine learning model to predict vascular disease.

ML is a class of AI algorithms that applies statistical characterization methods to manually extracted features (generally numerical) based on various image properties, i.e., brightness, contrast, and texture. A series of studies have been conducted for TC using different medical organs and medical imaging modalities [15,16,17,20,37,38,156]. Figure 13 shows a typical ML model to predict vascular disease. It has two components, namely, an offline training system and an online prediction system. Data acquisition can be seen for four kinds of images, namely carotid, coronary, renal, and pulmonary vasculature. The machine system can be executed for any kind of vascular disease, as shown by the “vascular artery switch”. The offline system consists of offline feature extraction, where the grayscale features are extracted. These features undergo training model generation using (i) training-based grayscale features, (ii) gold standard labels, and (iii) classifier type. The prediction system consists of testing-based grayscale features, which are then transformed by the training model to predict the vascular disease risk label type, which is a two-class system (disease vs. controls). Several examples of ML systems for different applications have been developed previously [157]. The result of the predicted system is sent to the performance evaluation system, which uses the result of the predicted system and the gold standard to figure out the receiving operating curves (ROC).

### 5.1. AI-Based Tissue Characterization for Pulmonary Disease Diagnosis in COVID-19

The intensity of the severity of lung infection due to COVID-19 is currently being quantified by radiologists through various imaging modalities, including X-ray, MRI, CT, and ultrasound [40]. In this regard, different schools of thought envision AI-based automated solutions for the detection and quantification of the severity of COVID-19-induced ARDS from lung images. There are two parts to this analysis: (a) segmentation of the lung using model-based techniques [158], and (b) classification of COVID-19 disease in these segmented lungs. For these tasks, AI algorithms are further divided into two categories, machine learning (ML) [159] and deep learning (DL) [160,161,162].

**Figure 14:**
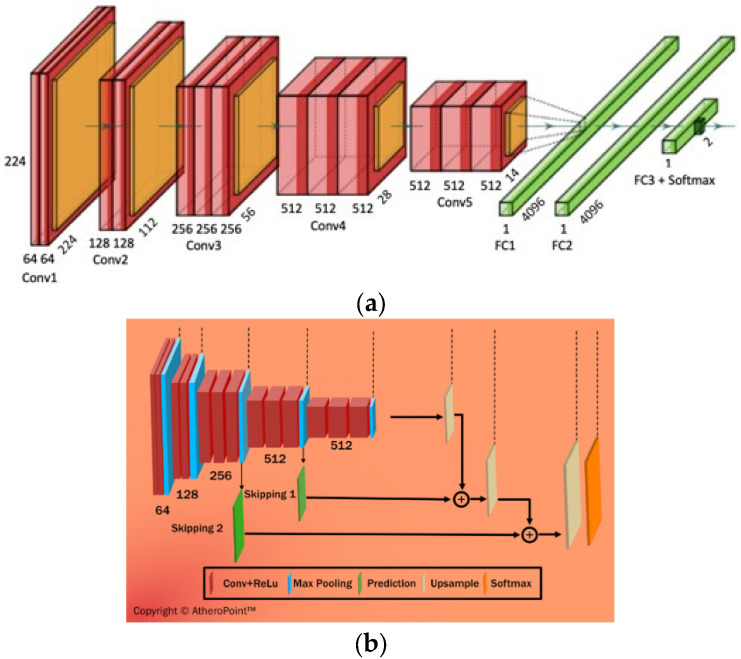
(**a**) DL-CNN model for characterization, (**b**) DL-FCN model for segmentation [44].

DL is a class of AI algorithms that uses a neural network to mimic the visual cortex of the brain for segmentation and AIbTC [163]. It has been found that although DL models are costly in terms of computational time and storage, they are more accurate than ML strategies. The DL characterization module is shown diagrammatically in Figure 14a, while the segmentation module is shown in Figure 14b. DL-CNN uses a sequence of convolutional, ReLu, and pooling layers to extract features that are then passed to fully connected layers to perform characterization [164,165]. On the other hand, DL-FCN models use upsampling and skipping of layers to perform semantic segmentation [166]. CNN is a neural network model that extracts picture representations. It examines an image’s original pixel data, trains a model, and automatically extracts features for better categorization. Fully Convolutional Networks segment semantic data, and solely use convolution, pooling, and upsampling.

**Figure 15:**
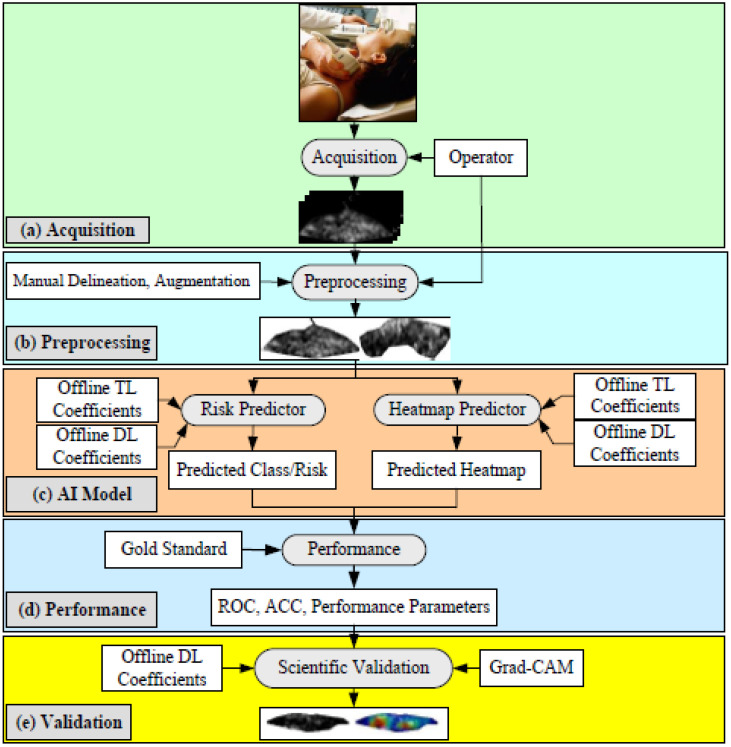
Deep learning model to predict vascular disease using AIbTC [44].

Figure 15 depicts the AIbTC architecture that has been proposed to scan the internal carotid artery in the cloud domain. It is comprised of the following five components: (a) image capture, (b) preprocessing, (c) artificial intelligence-based models, and (d–f) performance assessment and verification. In order to acquire the plaque region of interest, these scans are first normalized and then manually delimited in the pre-processing phase (ROI). The augmentation block was included as a part of the pre-processing phase block, as the cohort size was quite modest. This block assists in determining whether plaques are symptomatic or asymptomatic. This is accomplished by having trained AIbTC models perform a transformation on the image of the testing plaque.

Altogether, seven schools of thought (SOT) have been used extensively in AIbTC area. For convenience, the groups have been named the Beijing group, Changsha SOT, Wuhan SOT, Macau SOT, Trento SOT, Bethesda SOT, and Molise SOT. The Beijing SOT has used several models of DL, i.e., ResNet, VGGNet, DenseNet, and UNet (architecture details are shown in Appendix B) for multiview fusion, video-based real-time prediction, and semi-quantitative prediction of COVID-19-induced ARDS severity [167,168,169,170,171]. The Changsha SOT has used a biomarker-based model for severity detection in 3D lung abnormalities. The DL models used were Resnet34 with logistic regression and Dense UNet for CT, MRI, and ultrasound [172,173]. The Wuhan SOT applied DL models extensively to CT, MRI, and ultrasound lung images for characterization [174,175,176,177]. The Macau SOT used a combination of DL and ML algorithms, i.e., Resnet with Gradient Boosting, for the characterization of ARDS severity [178]. The Trento SOT used ML models, such as the Hidden Markov Model, SVM, and Random Forests, for ARDS detection and characterization, i.e., pleural line identification, automatic severity assessment, and exploration of severity-related features [179,180]. The Bethesda SOT used a combination of DL and TL for ARDS characterization from different imaging modalities [181,182]. The Molise SOT used a combination of ML, DL, and TL models for the classification of COVID-19 disease [183,184,185]. The various DL model architectures are explained in Appendix B.

### 5.2. AI-Based Tissue Characterization for Renal Disease in COVID-19

As previously stated, COVID-19 damages the kidneys both directly and indirectly. In the direct form of damage, the proximal tubular cells of the kidney are directly damaged due to the intrusion of the SARS-CoV-2 virus [186]. In the indirect invasion, COVID-19-induced cytokine storms and the subsequent hypercoagulable state of tissue factors lead to renal ischemia and cortical necrosis [187]. These abnormalities are easily observed in renal images and diagnosed by radiologists. In recent years, AI-based measures, especially DL models [188] and model-based imaging [189], have increasingly found prominence in the detection of abnormalities [190] and in the segmentation of kidney images [191]. Several of these works are discussed below.

Hermsen et al. [192] used the DL model for automated segmentation of five structures within the kidney, i.e., glomeruli, proximal tubuli, distal tubuli, arterioles, and capillaries, with a high degree of accuracy. The accuracy of DL primitive FCN, U-net, and M-FCN appears to be better. Except for capillaries and arterioles, which scored about 30% lower, the majority of classes scored close to 90%. Kolachalama et al. [193] used the DL model to classify different stages of chronic kidney disease (CKD), serum creatinine, and nephrotic-range proteinuria. The DL model’s accuracy and area under curve were better than in previous models. Nephrologists employ kidney length, volume, cortical thickness, and echogenicity to assess kidney damage. The very short renal length (8 cm), whitish cortex, and contracted capsule contour indicates permanent kidney failure. In the aforementioned study, the ResNet model was discussed as having an accuracy of 87%. Kuo et al. [194] used transfer learning for the identification of CKD status using kidney ultrasound images. This is the first study to link the retina and kidney using an AI-based Deep Learning Accelerator (DLA), showing the potential of retinal pictures to diagnose and screen CKD illness in the population. DLAs could be implemented into retinal cameras as a complement to serum creatinine and estimated glomerular filtration. Multimodality imaging plays a vital role in better disease detection and is very helpful in monitoring and validating the clinical results. This requires image registration [195,196,197,198].

### 5.3. AI-Based Tissue Characterization for Carotid/Coronary Disease Diagnosis in COVID-19

COVID-19 induces endothelial barrier interruption and causes harm to the vascular wall due to major action on angiotensin II type 1 (AT1) receptors. The degradation of the epithelial layer leads to the acceleration of atherosclerosis disease [199]. As atherogenesis sets in, low-density lipoprotein (LDL) cholesterol accumulates along the artery walls, leading to their hardening [200]. Other materials, such as macrophages and fibrous tissue, enter the arterial wall, leading to the formation of a complex necrotic core representing the plaque with a vulnerable thin fibrous cap [201]. With time, the fibrous tissue may rupture, leading to thrombosis and subsequent stroke [202]. Thus, imaging-based characterization is necessary for stroke risk estimation [203,204]. Accordingly, an iterative approach to examining epithelial cellular health can be applied using AIbTC and classification [189,205] in medical imaging.

#### 5.3.1. The General Framework for PTC using CNN

**Figure 16:**
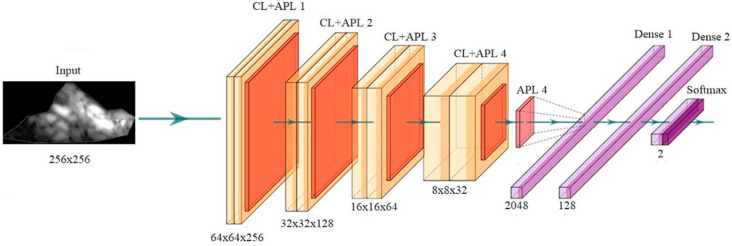
The general structure of CNN architecture (courtesy of AtheroPoint™, Roseville, CA, USA) [206,208].

DL with a convolutional neural network can be used to improve features or obtain useful information from images. Figure 16 shows how the extraction of features can be carried out in two ways, either a 1D or a 2D way. CNN technology has four main features: max pooling, convolution, non-linearity, and classification [206,207].

Early AIbTC screening is vital for plaque identification and risk stratification [33]. The plaque area is measured in terms of carotid intima–media thickness (cIMT) and total plaque area [209]. The plaque type is generally characterized as either symptomatic or asymptomatic. With regard to cIMT and TPA measurement, the plaque area is first segmented and corresponding measurements are made. Several DL models have been used to segment the plaque area accurately. Biswas et al. [210] developed a single-stage DL model for segmenting plaques with significantly lower bias concerning contemporary methods in the same domain. Guadrado et al. [211] used a similar strategy to compute the TPA. In 2020, Biswas et al. [212] developed a two-stage DL model for cIMT and TPA measurement, with even better results. Several recent techniques using DL have been developed for area measurements using Jain et al. [32,213,214]. Numerous AIbTC methods are used for stroke risk assessment [35,215]. In the area of plaque characterization, Lekadir et al. [216] used the AIbTC model for determining the composition of plaques with fair accuracy. In 2020, Skandha et al. [217] used the DL-CNN model for the characterization of plaques with better accuracy.

**Figure 17:**
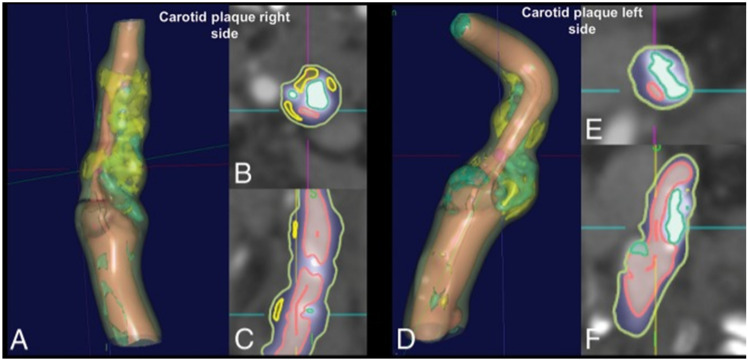
Carotid plaque with bilateral intraparenchymal hemorrhage [218]. (Carotid plaque right side, (**A**): 3D view, (**B**): top view, (**C**): side view, Carotid plaque left side (**D**): 3D view, (**E**): top view, (**F**): side view.)

Saba et al. [218] demonstrated a plaque characterization approach using CT scans on symptomatic subjects with bilateral intraplaque hemorrhage (Figure 17). The authors measured plaque components such as calcification (value ≥ 130 HU), mixed plaque (values ≥ 60 and <130 HU), lipid (value ≥ 25 and <60 HU), and intraplaque hemorrhage (value < 25 HU). The authors showed that the IPH/lipid ratio was higher on the symptomatic side (0.596 versus 0.171, *p* = 0.001).

In the advanced stages of COVID-19 infection there is an increased production of cytokines, inducing organ damage; medical imaging with AI can help in the advanced diagnosis of the recent pathophysiology of the patient [219]. The several DL-based tools discussed above can help in faster detection of vascular abnormalities with a lower risk of infection.

#### 5.3.2. Coronary and Carotid Plaque Tissue Characterization Using Machine Learning

ML and DL methods for the carotid plaque tissue characterization (PTC) approach [24,220] are needed to delineate how high the risk of CVD is in mild COVID-19 patients versus severe COVID-19 patients. In the field of clinical imaging, ML has been used to implement popular classifiers such as random forest (RF), support vector machine (SVM), decision tree (DT), and AdaBoost. Due to changes in US, PTC can meet diagnostic and therapeutic needs while keeping costs down. Saba et al. [221] used a PCA method based on polling in an ML framework to pick the most important traits for better performance. The majority of cardiologists use ML to determine the risk of CHD before stenting and percutaneous coronary intervention [35]. This study used a method that used intravascular ultrasonography (IVUS), greyscale plaque morphology, and cIMT to measure the risk of CVD.

**Figure 18:**
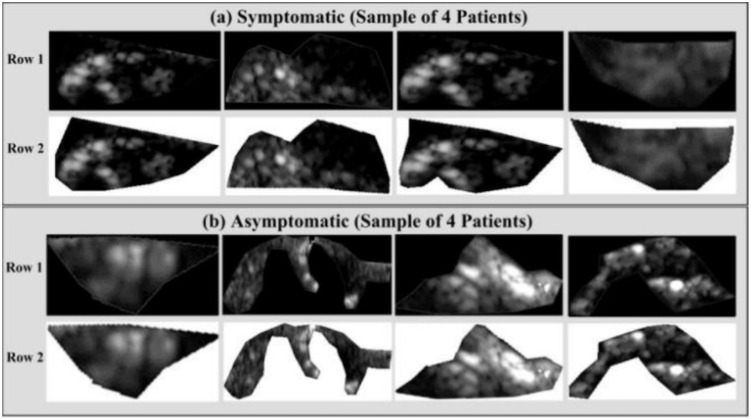
Delineated plaque in B-mode US: (**a**) symptomatic plaque and (**b**) asymptomatic plaque (Courtesy of Atheropoint^TM^, Roseville, CA, USA) [44].

Using the AIbTC of symptomatic and asymptomatic plaque from US images, vascular radiologists can promptly elucidate patient diagnoses. Acharya et al. [31] looked at 346 pictures of US plaques, of which 196 showed symptoms and 150 showed no symptoms. Figure 18a,b shows two examples of plaque with symptoms and plaque with no symptoms. The photos were first processed to eliminate noise, then a discrete wavelet transform (DWT) was used to pull out the features.

In the framework of ML, a wide range of studies have been carried out to investigate various aspects of risk assessment for CTAD and COAD [222,223,224]. In addition, ML was used to identify people with COAD by assessing the greyscale features of left ventricular ultrasound data [225]. Recent research has resulted in the development of a method for forecasting the risk of COAD that is based on DL and makes use of the carotid artery as the gold standard [44,45,226,227].

#### 5.3.3. Plaque Tissue Characterization Using Deep Learning

**Figure 19:**
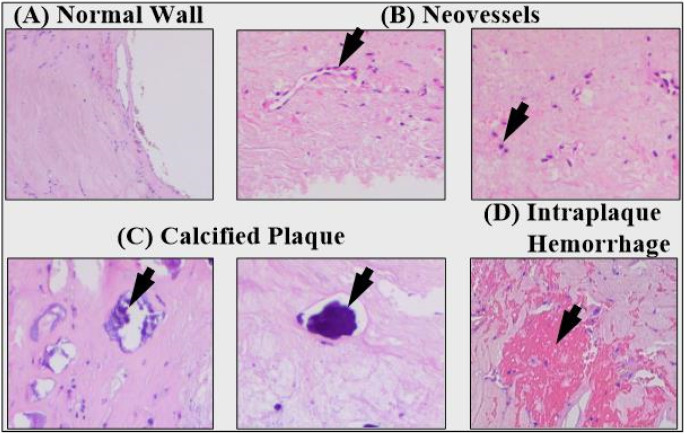
Different plaque components depicted in pathological pictures: (**A**) healthy wall, (**B**) neovessels, (**C**) calcified plaque, and (**D**) interplaque hemorrhage (courtesy of Dr. Luca Saba, U of Cagliari, Italy) [222].

The engagement and movement of smooth muscle cells (SMCs) from the media layer to the intima layer are necessary steps in the development of a plaque. The plaque generation process is characterized by the migration of SMCs from the media layer into the intima layer, where they consolidate to form the majority of the cellular auxiliary matrix. These SMCs from the media layer have the potential to infiltrate the surface, where they can form a layer known as the fibrous cap. This layer possesses an elastic quality that protects it from cracking, and it was created by these SMCs. The risk of producing a fracture, on the other hand, rises as the layer in question becomes more rigid. Inflammatory cells are responsible for destroying SMCs, which help to reinforce and stabilize the cap. Different plaque components are depicted in the pathological pictures shown in Figure 19. The images of plaque indicate a healthy wall with neo-vessels, calcified plaque, and intraplaque bleeding.

The stiffness index of the cap is what influences the likelihood of a plaque breaking apart. Therefore, measuring stiffness as part of a stroke risk assessment is quite important. This is related to the process of distinguishing between hard tissues and soft tissues [43].

#### 5.3.4. Generalized Transfer Learning for AI-Based Tissue Characterization

**Figure 20:**
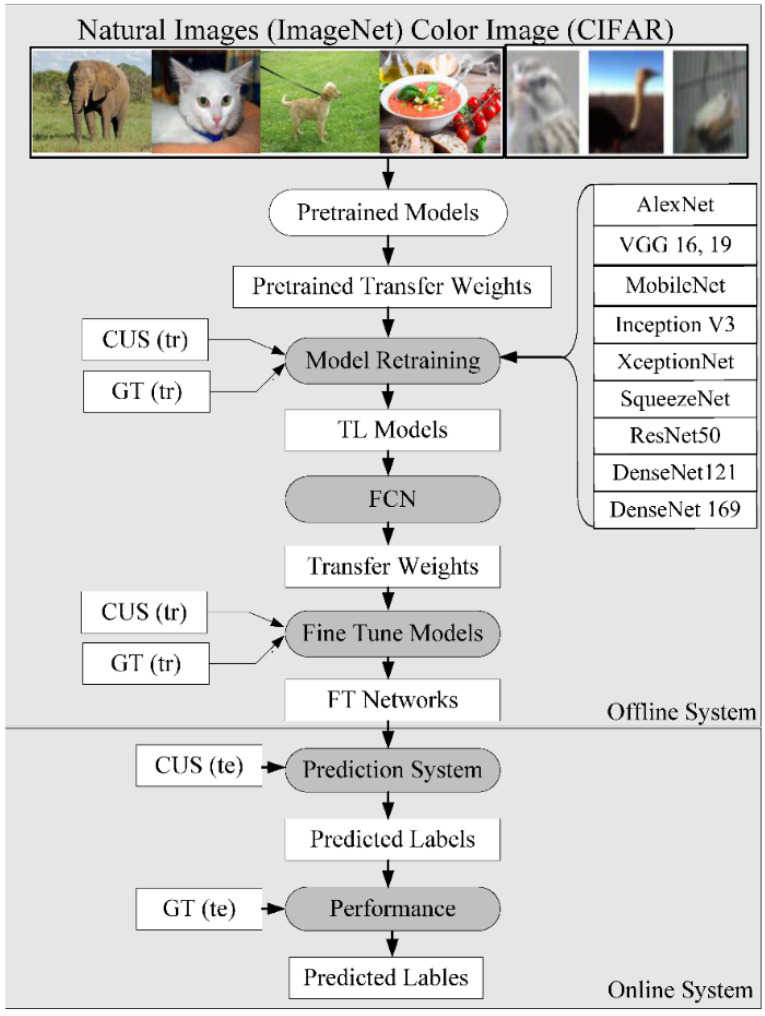
Transfer learning model to predict vascular disease [44].

**Figure A2:**
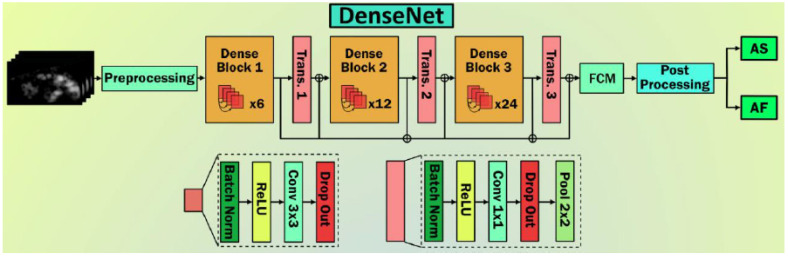
The general structure of Densenet architecture (courtesy of AtheroPoint™, Roseville, CA, USA) [44].

**Figure A3:**
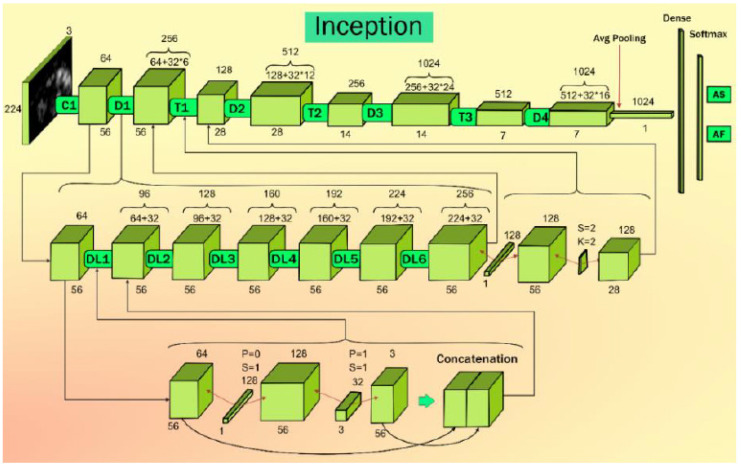
The general structure of the InceptionV3 architecture [44].

**Figure A4:**
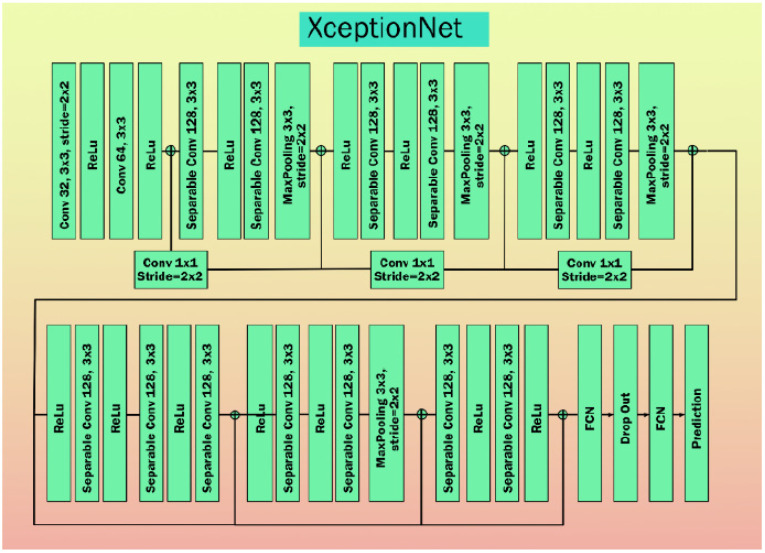
The general structure of XceptionNet architecture [44].

**Figure A5:**
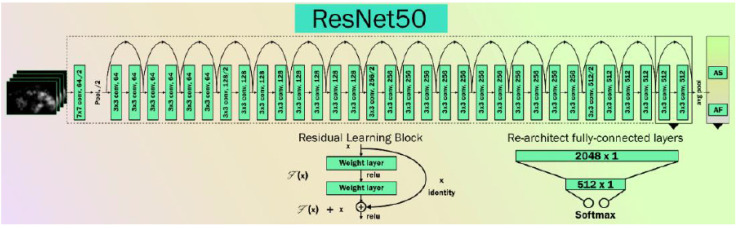
The general structure of Resnet50 architecture [44].

**Figure A6:**
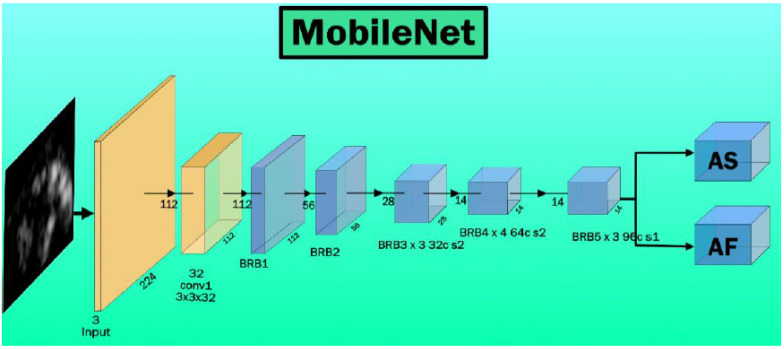
MobileNet Architecture, BRB: bottleneck and residual blocks [44].

The transfer learning (TL) architecture for PTC is shown to be more efficient [19,44,227,228]. This is because the initial weights are not computed, and are instead taken as pretrained weights to start the training and prediction process. An example can be seen in Figure 20. We demonstrate the use of three kinds of fundamental architecture, such as VGG, DenseNet, and ResNet; each of these has different versions of the base framework. Thus, the VGG group has VGG-16 and 19, DenseNet (Figure A2) architectures have DenseNet121 and 169, and ResNet architectures have ResNet50 (Figure A5) and 101. The core change between these versions is the number of neural network layers. It is important to note that the latest architectures such as MobileNet (Figure A6) and XceptionNet (Figure A4), Inception V3 (Figure A3) are more modern, and are well-adapted in the AI industry, showing faster optimization paradigms. Various DL models with descriptions are shown in Appendix B.

## 6. Discussion

### 6.1. Principal Findings

In this review, we have focused on the deep causes of vascular damage, whether in the pulmonary, renal, carotid, or coronary vessels. Thus, it is vital to understand the vascular pathophysiology in these four vascular territories. This special report has helped with understanding of (i) the pathophysiology of vascular damage and the related role of radiological imaging, and (ii) AIbTC for understanding the vascular damage caused by COVID-19. Furthermore, this study provides new dimension in which to understand COVID-19 severity using different kinds of AI models in these vascular beds. Table 5 shows various studies depicting pulmonary, renal, coronary, and carotid artery vascular damage due to COVID-19.

Medical imaging methods such as MRI, CT, and ultrasound can be used for imaging the four kinds of vasculature infected by COVID-19 [229]. Several studies have shown that the extent of vascular damage and the characterization of COVID-19 can be facilitated using AI such ML, DL, and transfer learning paradigms [230]. Suri et al. presented several studies that focus on challenges in AIbTC (carotid, coronary) and present recommendations for improving AIbTC vs. control patients [32,66,156,224].

### 6.2. Benchmarking of Four Types of Vasculature Studies

Vascular damage to the renal, pulmonary, coronary, and carotid arteries due to COVID-19 has been linked in a few studies utilizing various imaging and non-imaging modalities such as MRI, CT, US, ECG, and X-ray, according to an overview of the data. AI’s function in the severity of vascular damage of four different kinds due to COVID-19 is rarely discussed in the literature. Only a few articles in the COVID-19 framework use the AIbTC model to describe the severity of vascular damage. Table 6 reports the benchmarking scheme for selected pulmonary, renal, coronary, and carotid artery AIbTC abnormalities studies.

### 6.3. Pulmonary Vasculature Studies

Harmon et al. [231] proposed DL models that were trained on a diverse global cohort of 1,280 patients to localize forebrain pleura/lung parenchyma followed by classification of COVID-19 pneumonia. The DL model resulted in an accuracy of 85.50%, with 84% sensitivity, 0.94 AUC, and 93% specificity. Estépar [232] presented a CNN model and their interpretation of the pulmonary vasculature observations of 1,024 patients. Pulmonary function test (PFT) pattern identification and diagnosis were 100% and 82% accurate, respectively, using the automated method.

**Table 5:** Comparison of pulmonary, renal, carotid, and coronary artery damage due to COVID-19.

SN	COVID-19 Attributes	Pulmonary	Renal	Coronary	Carotid
1	Viral invasion	ACE2 receptors on surface of type 2 pneumocytes	ACE2 receptors on the surface of glomerular cells, tubular epithelium, and podocytes of kidneys.	Myocytes [233]	ACE2 receptors
2	Manifestations	ARDS	Acute kidney injury, acute tubular necrosis, cortical necrosis, and renal ischemia, tissue abnormalities.	Plaque variability, abnormality in blood flow, Myocardial ischemia, myocarditis, and heart failure	Atherosclerotic plaque vulnerability and promotes a thrombogenic environment.
3	Systemic abnormalities (i.e., DM, HTN, ARDS, CVD)	Primary	Secondary	Primary and secondary	Primary and secondary
4	Anticoagulants	May be beneficial [234].	Not beneficial [235]	Beneficial [236]	Beneficial [236]
5	Imaging Modalities	CT shows subpleural and peripheral areas “ground-glass opacities” and consolidation [236].	CT, US, and MRI	CT, US, MRI, and X-ray	CT, US, and MRI
6	AI Models	ML [237], DL [238], HDL [46]	ML [239], DL [240], HDL [241]	ML [204], DL [242], HDL [243]	ML, DL, HDL
7	Classifier Types	SVM, DT, CNN, RF	SVM, DT, CNN, NB	SVM, DT, CNN, RF	SVM, DT, CNN, RF, NB
8	Drugs commonly used in COVID-19 may induce these conditions	Remdesivir is a prodrug for its action it metabolizes to Remdesivir triphosphate	Remdesivir is a prodrug for its action it metabolizes to Remdesivir triphosphate. Both Remdesivir and its active metabolite eliminate renal (i.e., 74%). AKI with this drug may be transient. Hence it is not advised in patients with eGFR < 30 mL/min per 1.73 m^2^ [244].	Chloroquine phosphate, hydroxychloroquine sulphate and azithromycin usage individually or in combination may increase in QTc interval prolongation and torsades de pointes or ventricular arrhythmias [245,246].	Chloroquine phosphate, hydroxychloroquine sulphate, and azithromycin

Li et al. [247] demonstrated the DL model for finding lung lesion segmentation via CT scan using 4,332 patient images utilized for the analysis. The model used a combination of the CNN algorithm for feature extraction followed by classification using an SVM extractor. The model showed a high sensitivity of 90% (95% confidence interval (CI): 83% to 94%) and a high specificity of 96% (95% CI: 93% to 98%). For COVID-19 and community-acquired pneumonia, the areas under the receiver operating curves were 0.96 (95% CI: 0.94, 0.99) and 0.95 (9% CI: 0.93, 0.97), respectively.

Saba et al. [238] proposed six models to differentiate between COVID-19 pneumonia (CoP) and non-COVID pneumonia. A 100-patient dataset was used for the purposes of experimentation. Three kinds of AI models were used, two conventional ML (k-NN and RF), two TL (VGG19 and InceptionV3), and two DL models (CNN and iCNN). For CT lung characterization, a K10 cross-validation (90% training, 10% testing) protocol was used on an Italian cohort of 100 patients with CoP and 30 patients without CoP. The study results showed that K-NN, VGG19, IV3, CNN, and iCNN all had accuracies in the range of 74.58% to 96.74%; the associated AUCs were 0.74, 0.94, 0.96, 0.98, 0.99, and 0.99, respectively, all having *p*-values = 0.00001.

Agarwal et al. [46] demonstrated a novel AI-based method for COVID-19 disease classification, characterization, and severity measurement in lung CT scans on an Italian cohort. The presented work explains a two-stage CADx system involving (i) segmentation and (ii) classification. The classification system included a CNN, five transfer learning algorithms, random forest, a decision tree, and ANN soft classifiers. The system included block imaging, bispectrum analysis, and entropy analysis for lung AIbTC. Diagnosis odds ratio, receiver operational parameters, and CADx system statistics were used to evaluate the performance. CNN and Random Forest were the top soft classifiers, with 99.41 ± 5.12% accuracy and AUC 0.991, *p* < 0.0001, respectively. The characterization system showed the most accurate color-coded probability maps in COVID-19 patients’ inferior lobes.

### 6.4. Renal Vascular Studies

Tseng et al. [248] analyzed the relationship between cardiac surgery and acute kidney damage (CSA-AKI). There can be a significant complication known as cardiac surgery-associated acute kidney damage (CSA-AKI), which can lead to an increased risk of death as well as an increased risk of morbidity. A total of 671 individuals who were scheduled to have heart surgery were included in the study. Logistic regression, support vector machine (SVM), random forest (RF), extreme gradient boosting (XGboost), and ensemble (RF + XGboost) were among the ML algorithms used for analysis. The effectiveness of these models was assessed by calculating the AUC. RF exhibited the greatest AUC of 0.839, 95% accuracy, and CI 0.772–0.898 when compared to the efficacy of the single model that most accurately predicted the outcome; however, the AUC of the ensemble model (RF + XGboost) was even greater than that of the RF model alone, with 0.843, 95% accuracy and CI 0.778–0.899.

Zang et al. [249] developed an AI-based pulse-coupled neural network (PCNN) for enhancing ultrasonic image information, and this algorithm was compared against the histogram equalization and linear transformation methods. The model was built using a CNN-based algorithm. This was later used in hospital settings to aid in the ultrasonic image diagnosis of 31 patients who were suffering from acute sepsis in conjunction with an AKI. The condition of each patient was diagnosed based on (a) ultrasound image performance, (b) the change in renal resistance index (RRI), (c) the ultrasound score, and (d) an analysis of the ROC.

Ying et al. [250] proposed a PCNN method for the diagnosis of severe sepsis complicated by AKI using an ultrasonic image. Their study explains their CNN-based ultrasonic image enhancement technique, which was later compared with the histogram equalization and linear transformation algorithms. Twenty patients with severe sepsis and AKI were then diagnosed using ultrasonic imaging. The algorithm resulted in an AUC of 0.78.

Bouteldja et al. [251] proposed a DL-based CNN model for verification of vascular abnormalities in the kidney using 60 renal AIbTC scans. Their paper differentiates six important renal structures, including the glomerular tuft, the glomerulus, Bowman’s capsule, the tubules, the arteries, the arterial lumina, and the veins. The implemented model shows 81% accuracy and 0.80 AUC.

Kalisnik et al. [252] explained an ML model using an SVM classifier for early detection of AKI after cardiac surgery with a cohort of 288 patients. After cardiac surgery, AKI was detected with an area under the curve of 88%, a sensitivity of 78%, a specificity of 78.1%, and an accuracy of 82.1%.

### 6.5. Coronary Vasculature Studies

Çolak et al. [253] proposed the prediction of coronary artery disease using the ANN model. Their experimentation included 124 consecutive patients with CAD (at least one coronary stenosis > 50% in main epicardial arteries). In total, 113 patients with normal coronary arteries (group 2) served as angiographic controls. Their ANN architecture used a multi-layered perceptron. The ANN models were trained on 237 training (n = 171) and testing (n = 66) record sets. The proposed model showed 71% sensitivity, 76% specificity, and 80% accuracy.

Correia et al. [254] presented an ML-based algorithm for detecting coronary disease in individuals having chest pain and compared it to the classical statistical model, using 962 chest pain patients. An ML method and a classical logistic model were created utilizing the first two-thirds of patients. The remaining one-third of the patients had these two prediction techniques tested. The final logistic regression model had just 5% significant variables. The sample was 59 ± 15 years old, 58% male, and 52% had coronary disease. The model had nine independent predictors. All predictor candidates were in the ML algorithm. In the test sample, the ML algorithm’s ROC curve for coronary disease prediction was 0.81 (95% CI = 0.77–0.86), identical to the logistic model (0.82; 95% CI = 0.77–0.87), *p* = 0.68.

Cheng et al. [255] suggested a ANN model that achieved satisfactory performance in the prediction of MACE in patients who required coronary artery syndrome (CAS) treatment. The study used 317 patients for the experiment. The accuracy of the model was 80.76%. When neurologists recommend patients to cardiologists, as well as before patients are treated by cardiologists, a model ANN can be useful for detecting high-risk patients who have CAS. It might also serve as a communication reference when patients are referred to cardiologists.

### 6.6. Carotid

Jain et al. [256] proposed an AI model for the examination of atherosclerotic plaques in the internal carotid artery. These plaques may rupture and cause embolism of cerebral blood vessels, resulting in a stroke. A total of 970 ICA B-mode US pictures from 99 high-risk patients were incorporated into the database. Difference area thresholds of 10 mm^2^ between AI and GT yielded AUC values of 0.91, 0.911, 908.9, and 905, (CE-loss models) and 0.98 (AI-loss models), respectively, for DSC-loss models, all with *p*-values of less than 0.001. An AI plaque area and a GT plaque area had correlations of 0.98, 0.96, 0.97, 0.97 (for CE-loss models), whereas a correlation of 0.98, 0.98, 0.97, 0.97 (for GT plaque area) was found in the study for DSC-loss models. Online plaque segmentation takes less than a second. The HDL and SDL models behave equally, confirming our hypothesis. SegNet-UNet had the best performance.

Skandha et al. [257] proposed a plaque characterization approach utilizing CT images on symptomatic participants with bilateral intraplaque bleeding (Figure 18). The authors examined plaque components such as calcification (value ≥ 130 HU), mixed plaque (values ≥ 60 and <130 HU), lipids (value ≥ 25 and <60 HU), and intraplaque bleeding (value < 25 HU). The authors showed that the IPH/lipid ratio was greater on the symptomatic side (0.596 versus 0.171, *p* = 0.001).

In the benchmarking section, we discuss the pathophysiology of pulmonary, renal, coronary, and carotid vasculature damage in patients with COVID-19, as well as the current evidence for these consequences. All four mentioned organs are common in individuals with COVID-19 who are in severe condition, and all are linked with a high fatality rate. The role of AI methods such as ML, DL, and transfer learning paradigms is seen in various mentioned studies, and can aid in determining the level of vascular damage and characterizing patients’ COVID-19 condition. However, no studies were able to explain bias in AI systems.

**Table 6:** Benchmarking scheme for four types of COVID-19 vascular damage.

	C1	C2	C3	C4	C5	C6	C7	C8	C9
SN	Authors, Citation and Year	Vascular Type	IM	AI Type	Patient Dataset	Feature Selection	Classifier Type	Accuracy (%)	AUC [0-1]
1	Harmon et al. [231] (2020)	Pulmonary	CT	DL	1280	NR	NR	85.50	0.94
2	Estépar [232] (2020)	Pulmonary	CT	DL	1024	PCA	CNN	92.00	0.80
3	Li et al. [247] (2020)	Pulmonary	CT	DL	4332	SVM	CNN	94.00	0.96
4	Saba et al. [238] (2020)	Pulmonary	CT	DL	130	SVM	CNN	74.58	0.74
5	Agarwal et al. [46] (2021)	Pulmonary	CT	DL	30	DT	CNN	99.41	0.99
6	Tseng et al. [248] (2020)	Renal	CT	ML	61	SVM	RF + XGboost	79.00	87
7	Zang et al. [249] (2021)	Renal	MRI	DL	31	PCA	CNN	NR	NR
8	Ying et al. [250] (2021)	Renal	US	DL	20	NR	PCNN	NR	0.78
9	Bouteldja et al. [251] (2021)	Renal	US	DL	60	SVM	CNN	81.00	80
10	Kalisnik et al. [252] (2022)	Renal	CT	ML	288	SVM	RF	81.00	88
11	Çolak et al. [253] (2008)	Coronary	CT	DL	237	NR	CNN	97.08	0.92
12	Lee et al. [258] (2021)	Coronary	CT	DL	2985	NR	CNN	93.03	NR
13	Correia et al. [254] (2021)	Coronary	ECG	ML	962	SVM	LR	93.02	0.93
14	Upton et al. [243] (2022)	Coronary	CT	DL	832	NR	CNN	92.07	0.93
15	Gao et al. [259] (2022)	Coronary	US	ML	539	RF	LR	89.05	NR
16	Cheng et al. [255] (2017)	Carotid	US, CT	DL	317	NR	ANN	80.76	0.80
17	Skandha et al. [227] (2020)	Carotid	CT	DL	1000	NR	CNN	95.66	0.95
18	Konstantonis et al. [260] (2020)	Carotid	US	ML	542	PCA	RF	98.39	0.98
19	Jain et al. [256] (2021)	Carotid	US	HDL	970	PCA	CNN	91.23	0.91
20	Skandha et al. [257] (2022)	Carotid	US	HDL	877	DT	CNN	99.78	0.99

IM: Imaging modality, AI: Artificial Intelligence, CT: Computer Tomography, US: Ultrasound, ECG: Electrocardiogram, ML: Machine Learning, DL: Deep Learning, HDL: Hybrid Deep Learning, SVM: Support vector machine, PCA: Principal Component Analysis, RF: Random Forest, CNN: Convolution neural network, LR: Logistic regression, MRI: Magnetic resonance imaging. NR: Not Reported.

### 6.7. A Special Note on Vascular Damage Due to COVID-19

**Figure 21:**
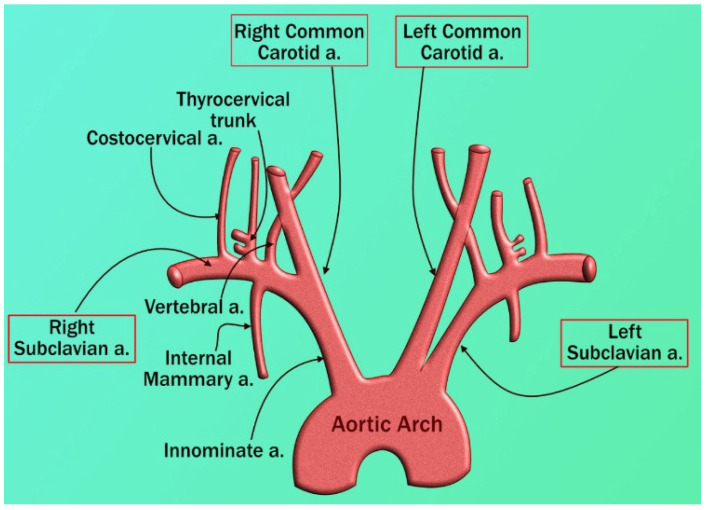
The inception of the left and right carotid arteries [271].

Vascular abnormalities increase the risk to both the heart and the brain [261]. This link has been extensively noted, as the genetic makeup of carotid and coronary arteries is similar [262,263]. The aortic arch, coronary artery, and carotid artery each have characteristics that are comparable to one another [264]. These arteries travel in opposite directions, even though each one branches off of a distinct major artery (Figure 21). Pathological changes such as polymorphonuclears, T-lymphocytes, histiocytes, monocytes, and mononuclear giant cells have been found in all specimens in the thrombus formation and all layers of vessels, in addition to endothelial proliferation and vascular endothelial, as well as varying degrees of collagen deposition and myofibroblastic proliferation [265,266].

These findings were found in conjunction with endothelial proliferation and vascular endothelium damage to the endothelium, which can lead to thromboembolism in the vasculature of the limbs and the aorta in addition to severe vascular events such as acute arterial hypoxia [267]. These promote LDL accumulation and oxidation, plaque formation, and arterial lumen narrowing [268,269]. Consequently, carotid artery disease has the potential to serve as a replacement biomarker for coronary heart disease in CVD patients who have COVID-19 [35]. COVID-19 is the agent that causes thrombosis in the veins and arteries, and is also the agent responsible for the unbalanced inflammatory state known as a cytokine storm, which affects endothelial cells as well [270].

### 6.8. Role of Anticoagulants in COVID-19

Research indicates that prophylaxis with low molecular weight heparin, such as fondaparinux, or oral anticoagulants, such as apixaban or rivaroxaban, may be considered in COVID-19 patients [272,273]. Heparin binds tightly to the spike proteins of the virus, resulting in impeded entry of SARS-CoV-2, downregulates cytokine storms, and reduces immune activation. Recent studies have shown that anticoagulants reduce mortality in COVID-19 patients. However, the role of anticoagulation in ARDS has been shown to not be beneficial [234]. Using anticoagulants and antiplatelets may be beneficial in the heart [236,274]. Further another study by Arnold F. et al. [235] shows using anticoagulants in the renal area is not beneficial.

### 6.9. Bias in Deep Learning Systems

The training model design step of DL algorithms is highly dependent on the sample size employed. Furthermore, lack of (i) clinical testing of AI techniques, (ii) scientific validation, (iii) not satisfying the gold standard, (iv) comorbidities in sample sets, (v) lack of big data configuration, and (vi) not judging the proper disease severity ratio can all lead to bias in an AI. As a result, when COVID-19 results in vascular damage (or related risk factors) are examined as inputs to an AI model, it is critical that the AI model be stable and accurate and have minimal AI bias [37,275,276,277,278]. It may be noticed that the database contains geographically specific patient characteristics. As a result, the model may produce deceptive positive or negative findings when used for other regions due to the introduction of bias into the model [279,280].

### 6.10. Strengths, Weakness, and Extensions

The main strength of the current study is the identification of vascular damage in the pulmonary, renal, coronary, and carotid arteries due to COVID-19. DL offers better training and risk prediction due to superior non-linear adjustment between the covariates and the gold standard. In addition, the system offers better coverage of covariates such as image modalities of CT, US, MRI, and X-ray. Furthermore, CNN represents a very powerful approach to DL system design for AIbTC vascular damage risk prediction. Lastly, the DL system is generalized, which can be altered by adding more covariates and comorbidities, thereby designing a multiclass system [19]. While DL offers strengths, it needs to be ensured that the system is optimized. This requires several iterations of systematic hit-and-trial attempts to achieve optimal DL solutions. Furthermore, a DL system requires a solid gold standard for CT lesion annotations for vascular damage and their respective gold standard collection in cohorts, which requires a considerable length of time and costs.

Lastly, DL systems are susceptible to AI bias due to overperformance in terms of accuracy and lack of interpretability along with clinical evaluation. In terms of extensions, superior DL systems can be designed using ensemble-based methods. Big data can be considered as an option for improving DL systems by using more sources of data and a larger sample size. Even though our strategy adapted standardized engineering protocols for AI-based tissue characteristics for vascular damage due to COVID-19, a more exhaustive search could be adopted using Embase, Medline, and The Cochrane Library.

The DL system can be improved by adding augmentation designs, should the cohort size be small. Note that a new wave of pruning then needs to be incorporated into the DL system for smaller-size training storage models [281] and evolutionary methods [282]. Lastly, integration of advanced image processing methods can be integrated for better loss function designs [283].

## 7. Conclusions

This is the first special report of its kind to focus on vascular damage due to COVID-19 along with the role of radiological imaging of the pulmonary, renal, coronary, and carotid vessels. We demonstrated the pathophysiology of these four arterial vasculatures based on the hypothesis a link exists between vascular damage and COVID-19 severity. We showed the role of radiological imaging techniques such as MR, CT, X-rays, and US for acquiring vascular data, which can then be used in the design of AIbTC systems. It was this AIbTC that was used for risk stratification of COVID-19 damage in pulmonary, renal, coronary, and carotid arteries. In terms of AI, this is the first kind of special report to demonstrate that, for case studies of four vascular damage types due to COVID-19, AIbTC models such as the machine learning, deep learning, and transfer learning paradigms can aid in determining the level of vascular damage and characterizing patients’ COVID-19 condition. In addition, the study focuses on obstacles and provides ideas for strengthening AI-based architectures for risk stratification of COVID-19 severity.

Finally, the development of big data and artificial intelligence-based paradigms will likely lead to the development of future vascular risk assessment technologies that are more advanced.

## Figures and Tables

**Table 3:** The effect of COVID-19 on the coronary artery.

SN	Citations	PS	ME	Comorbidities	Outcome	Vascular Damage	Imaging Modalities	Treatment
1	Rudski et al. [138] (2020)	240	LBBM, OBBM	Hypertension	Palpitations, heart failure, chest discomfort, presyncope, and syncope are all possible manifestations of supraventricular and ventricular arrhythmias, which can arise throughout the subacute and chronic phases of the condition.	Myocardial damage	CT	NR
2	Rivero et al. [139] (2020)	01	LBBM, OBBM	NR	In the context of the pro-inflammatory response to the disease, cardiovascular disease (CV) may be a source of myocardial damage in people who have been infected with SARS-COVID-19.	An extreme elevation of the ST segment that resulted in myocardial damage or infarction has been observed on several occasions.	ECG	NR
3	Aghagoli et al. [140] (2021)	21	LBBM	Diabetes	Myocardial damage in people who have been infected with SARS-COVID-19.	Patients who require emergency coronary artery bypass grafting, repair of an aortic dissection, or replacement of the aortic valve	CT	NR
4	Gupta et al. [141] (2021)	180	LBBM, OBBM	High BMI	Inflammation persists over time and raises the risk of atherosclerotic disease as well as acute proinflammatory situations like the cytokine storm.	Patients with CAD who were treated with COVID-19 had a higher risk of myocardial damage.	CT	NR
5	Afshar et al. [142] (2021)	23	LBBM	Diabetes	The histological finding of diffuse endothelial inflammation in the submucosal arteries of the small intestine in COVID-19 patients is suggestive of the occurrence of microvascular small-bowel injury.	Myocardial Infarction	CT	NR
6	Catapano et al. [143] (2021)	12	LBBM	hypertension	Complications of the heart include things like myocarditis, acute coronary syndrome, and thromboembolic events, amongst others.	Endothelial Inflammation in the submucosal arteries of the small intestine	CT	NR
9	Aghagoli et al. [140] (2021)	21	LBBM	Diabetes	Myocardial damage in people who have been infected with SARS-COVID-19.	Patients who require emergency coronary artery bypass grafting, repair of an aortic dissection, or replacement of the aortic valve	CT	NR
10	Gupta et al. [141] (2021)	180	LBBM, OBBM	High BMI	Inflammation that persists over time and raises the risk of atherosclerotic disease as well as acute proinflammatory situations like the cytokine storm.	Patients with CAD who were treated with COVID-19 had a higher risk of myocardial damage.	CT	NR

PS: Patient size, ME: Method of evaluation, CVD: Cardiovascular Disease, LBBM: Laboratory base biomarker, OBBM: Office base biomarkers, NR: Not reported, CT: Computer Tomography, US: Ultrasound, MRI: Magnetic Resonance Imaging.

**Table 4:** Effect of COVID-19 on carotid vascular damage.

SN	Citations	PS	ME	Comorbidities	Outcome	Vascular Damage	Imaging Modalities	Treatment
1	Alkhaibary et al. [147] (2019)	01	LBBM	NR	Large-vessel occlusion due to COVID-19 infection	COVID-19 confers a significant risk of thromboembolic disease	CT	NR
2	Mohamud et al. [144] (2020)	06	LBBM	Hypertension	The COVID-19 virus has the potential to cause the rupture of susceptible atherosclerotic plaques, which can lead to thrombosis and acute ischemic stroke.	Patients with COVID-19 infection who have usual vascular risk factors are at a higher risk of LVO as a result of ICT.	MR	NR
3	Viguier et al. [148] (2020)	28	LBBM	Diabetes	The source of stroke should be sought by cervical CTA covering from the aortic arch to the vertex; nevertheless, common carotid arteries should not be overlooked, and the requirement for COVID-19 coagulopathy therapy should be stressed.	Acute ischemic stroke.	MRI	NR
4	Jud et al. [149] (2021)	01	LBBM	NR	Cardiovascular alterations may be caused by endothelial dysfunction.	Vascular reactivity and arterial stiffness may be altered in distinct ways by SARS-CoV-2.	CT	NR
5	Doo et al. [150] (2021)	02	LBBM	Hypertension	Edema of the cortex or sub cortex as a result of a breach in the blood–brain barrier	Carotid thrombosis with large ischemic stroke	MRI	NR
6	Qureshi et al. [151] (2021)	11	LBBM	Hypertension	COVID-19 effects on carotid strength	Increasing the rate of poor outcomes among patients with ischemic stroke and transient ischemic attack.	CT	NR
7	Ojo et al. [152] (2020)	221	LBBM	CKD, CVD	The consequence is more likely to occur in individuals who are older and who have a more severe disease; nevertheless, large-vessel occlusion is increasingly being documented in younger people.	Patients in COVID-19 who had a large-vessel ischemic stroke after sub occlusive acute restriction of the common carotid artery and thrombosis	NR	NR
8	Munjral et al. [153] (2021)	NR	LBBM, OBBM	BP, Diabetes	The importance of low-cost surrogate CVD tests, such as ultrasound screening of the carotid artery, can contribute to accurate AI-based risk assessment and the monitoring of atherosclerotic disease.	Highlighted the role that poor nutrition and vascular damage induced by SAR-CoV-2 played in causing damage to the brain and heart.	US	NR
9	Villadiego et al. [154] (2021)	04	LBBM	NR	The most distinctive feature of patients with COVID-19 is that they demonstrate severe hypoxemia, with arterial levels of oxygen (O2) tension even lower than 50 mmHg, and they do so without manifesting obvious signs of distress (dyspnea) or a significant increase in the rate at which they are breathing.	Vascular damage induced by SAR-CoV-2	NR	NR
10	Crispy et al. [155] (2022)	15446	LBBM, OBBM	Diabetes, CVD	Endothelial Dysfunction results carotid alternation	Carotid Revascularization	US	NR

PS: Patient size, ME: Method of evaluation, CVD: Cardiovascular Disease, LBBM: Laboratory base biomarker, OBBM: Office base biomarkers, NR: Not reported, CT: Computer Tomography, US: Ultrasound, MRI: Magnetic Resonance Imaging.

## Data Availability

Not applicable.

## Abbreviations

**SN**	**Abb.**	**Definition**
1	ACC	American College of Cardiology
2	AIbTC	Artificial Intelligence-based tissue characterization
3	ARDS	Acute respiratory distress syndrome
4	ASCVD	Atherosclerotic cardiovascular disease
5	ANS	Autonomic nervous System
6	AUC	Area under curve
7	AI	Artificial Intelligence
8	BMI	Body mass index
9	CAD	Coronary artery disease
10	CAS	Coronary artery syndrome
11	CHD	Coronary heart disease
12	CKD	Chronic kidney disease
13	CT	Computed Tomography
14	CUSIP	Carotid ultrasound image phenotype
15	CV	Cross-validation
16	CVD	Cardiovascular disease
17	CVE	Cardiovascular events
18	DL	Deep learning
19	DM	Diabetes mellitus
20	EEGS	Event-equivalent gold standard
21	EMG	Electromyography
22	EC	Endothelial Cell
23	ER	Endothelium reticulum
24	FH	Family history
25	GT	Ground truth
26	HTN	Hypertension
27	HDL	Hybrid deep learning
28	ICAM	Intercellular adhesion molecule
29	VCAM	Vascular cell adhesion molecule
30	LBBM	Laboratory-based biomarker
31	MedUSE	Medication use
32	ML	Machine learning
33	MRI	Magnetic Resonance Imaging
34	NR	Not reported
35	NPV	Negative predictive value
36	NB	Naive Bayes
37	NO	Nitric Oxide
38	nOH	Neurogenic orthostatic hypotension
39	Non-ML	Non-machine learning
40	OBBM	Office-based biomarker
41	OH	Orthostatic hypotension
42	OxLDL	Oxidation of low-density lipoprotein
43	QTc	Chaotic heartbeat
44	PE	Performance evaluation
45	PPV	Positive predictive value
46	PCA	Principal component analysis
47	PRISMA	Preferred Reporting Items for Systematic Reviews and Meta-Analyses
48	PTC	Plaque tissue characterization
49	RA	Rheumatoid arthritis
50	PR	The period measured in milliseconds
51	RF	Random forest
52	ROS	Reactive Oxides Stress
53	RoB	Risk of bias
54	ROC	Receiver operating-characteristics
55	RNA	Ribonucleic acid
56	SCORE	Systematic coronary risk evaluation
57	SMOTE	Synthetic minority over-sampling technique
58	SVM	Support vector machine
59	TPA	Total plaque area
60	TC	Tissue Characterization
61	US	Ultrasound
62	tPA	tissue plasminogen activator

## Appendix A

### Acute Respiratory Distress Syndrome (ARDS)

Coronavirus infection causes lower levels of ACE2 to proliferate in the lung parenchyma cells. This is the outcome of the virus’s influence on the lungs; it causes a worsening of the accumulation of neutrophils, an increase in vascular permeability, as well as the generation of diffuse alveolar and interstitial exudates. As a consequence of this process, patients can develop pneumonia as well as acute respiratory distress syndrome (ARDS) [284]. Significant abnormalities in blood gas composition, which lead to low blood oxygen levels, are characteristic of ARDS. These abnormalities are the outcome of an oxygen and carbon dioxide imbalance that causes ARDS [285]. It has been established that this type of prolonged hypoxia leads to ischemia of the myocardium and damage to the heart [286,287]. In the brain, hypoxia speeds up the rate of anaerobic metabolism in the mitochondria of brain cells [288]. This causes cerebral vasodilation, edoema, and a reduction in blood flow, all of which are adverse effects of hypoxia. There is a chance of cerebral ischemia or acute cerebrovascular diseases such as acute ischemic stroke [288]. The sequence of events leading to ARDS is depicted in Figure A1.

An imaging technique is required in order to diagnose the anomalies in the lung, and x-rays and computer tomography are the two medical imaging techniques that play the most significant roles in the detection and diagnosis of COVID-19 [39,289]. CT has shown a great degree of sensitivity as well as reproducibility. Additionally, it is able to identify a variety of opacities, including ground-glass opacity (GGO), consolidation, and additional opacities [290,291], all of which are largely seen [292,293]. The outstanding potential exists for AIbTC systems to replicate conventionally established processes, which in turn enables quicker illness identification and diagnosis [291,294,295].

## Appendix B

### Appendix B.1. DenseNet Architecture

The vanishing gradient problem in deep neural nets is solved by the DenseNet design. Dense blocks are included in this design. After batch normalization (BN) and a pool of convolution layers with 3 × 3 filters to 1 × 1 filters, it uses “ReLU” activation on each layer.

Transition bricks are used to connect these thick blocks. Two-by-two filters with dropout and convolution layers, as well as pooling and convolution layers, are included in each transition block. DenseNet’s attractiveness is that it has parallel connections to keep the features from being lost.

### Appendix B.2. InceptionV3 Architecture

The DenseNet design overcomes deep neural nets’ vanishing gradient problems. This model features chunky building blocks. Every layer uses “ReLU” activation, and it has a pool of convolution layers that go from 3 × 3 filters to 1 × 1 filters. Finally, it uses batch normalization (BN).

These transition blocks are utilized to concatenate these dense blocks. Every transition block has dropout layers, convolution, and pooling layers, ranging from 22 to 11 filters. The fact that there are parallel connections within the DenseNet prevents any features from being lost, which is one of the network’s many appealing qualities.

### Appendix B.3. XceptionNet Architecture

Chollet et al. from Google suggested a change to the IV3 algorithm. As part of this update, the inception modules would be switched out for modified depth-wise separable convolution layers. This architecture is comprised of a total of 36 layers or strata. When compared to IV3, XceptionNet is quite lightweight and has the same number of parameters. The accuracy of this architecture in the top-one position is 0.790, and the accuracy in the top-five position is 0.945; both of these figures are higher than the performance of InceptionV3. Figure A4 shows an illustration of the architecture of the Xception-system.

### Appendix B.4. Resnet50 Architecture

The ResNet model can be utilized to tackle the vanishing gradient problem. Residual blocks include the ability to skip connections in their entirety. These connections bypass the training levels and go straight to the output. By skipping layers, the model is able to pick up more complicated patterns. In contrast to other models, this TL model makes use of data from CIFAR-10. Figure A5 illustrates ResNet. The architecture consists of two convolution layers that are each 3 × 3. The outputs and inputs of these pairs are merged before being sent to the pair that comes after them. Here, 64-512 filters are listed. Following the final 3 × 3 convolution layer, which has 512 filters, a flatten layer is used for vectorizing 2D features and the output is forecast with softmax activation.

### Appendix B.5. MobileNet Architecture

This was the first TensorFlow-based mobile computer vision model. The Flite database library is used for 28 layers. The architecture of MobileNet is depicted in Figure A6. Bottleneck residual blocks (BRBs), also known as inverted residual blocks, are employed in this model to reduce the number of training parameters.
